# Visualizing the mode of action and supramolecular assembly of teixobactin analogues in *Bacillus subtilis*†

**DOI:** 10.1039/d2sc01388f

**Published:** 2022-05-13

**Authors:** Michael A. Morris, Alexander Vallmitjana, Fabian Grein, Tanja Schneider, Melina Arts, Chelsea R. Jones, Betty T. Nguyen, Mohammad H. Hashemian, Melody Malek, Enrico Gratton, James S. Nowick

**Affiliations:** Department of Chemistry, University of California, Irvine Irvine California 92697 USA jsnowick@uci.edu; Laboratory for Fluorescence Dynamics, Department of Biomedical Engineering, University of California, Irvine Irvine California 92697 USA; Institute for Pharmaceutical Microbiology, University of Bonn, University Hospital Bonn Bonn 53115 Germany; German Center for Infection Research (DZIF), Partner Site Bonn-Cologne Bonn 53115 Germany; Department of Pharmaceutical Sciences, University of California, Irvine Irvine California 92697 USA

## Abstract

Teixobactin has been the source of intensive study and interest as a promising antibiotic, because of its excellent activity against drug-resistant Gram-positive pathogens and its novel but not yet fully understood mechanism of action that precludes drug resistance. Recent studies have demonstrated that the mode of action of teixobactin is more complicated than initially thought, with supramolecular assembly of the antibiotic appearing to play a critical role in the binding process. Further studies of the interactions of teixobactin with bacteria and its molecular targets offer the promise of providing deeper insights into its novel mechanism of action and guiding the design of additional drug candidates and analogues. The current study reports the preparation and study of teixobactin analogues bearing a variety of fluorophores. Structured illumination microscopy of the fluorescent teixobactin analogues with *B. subtilis* enables super-resolution visualization of the interaction of teixobactin with bacterial cell walls and permits the observation of aggregated clusters of the antibiotic on the bacteria. Förster resonance energy transfer (FRET) microscopy further elucidates the supramolecular assembly by showing that fluorescent teixobactin molecules co-localize within a few nanometers on *B. subtilis*. Fluorescence microscopy over time with a fluorescent teixobactin analogue and propidium iodide in *B. subtilis* reveals a correlation between cell death and binding of the antibiotic to cellular targets, followed by lysis of cells. Collectively, these studies provide new insights into the binding of teixobactin to Gram-positive bacteria, its supramolecular mechanism of action, and the lysis of bacteria that follows.

## Abstract 2

FRET microscopy experiments demonstrate supramolecular assembly of teixobactin molecules on *Bacillus subtilis*, providing further evidence that teixobactin is a supramolecular antibiotic.

## Graphical abstract

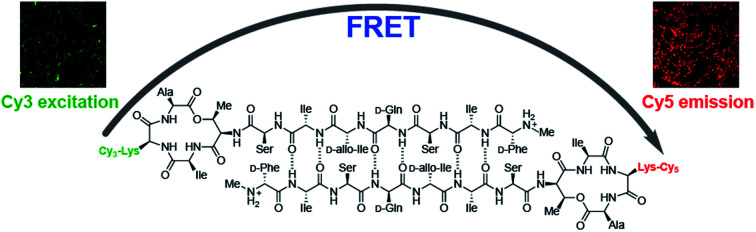

## Introduction

The peptide antibiotic teixobactin kills Gram-positive pathogens that are considered to be urgent and serious threats by the Centers for Disease Control and the World Health Organization.^1–3^ First reported in 2015, teixobactin has excellent potency against a variety of antibiotic-resistant bacteria, including methicillin-resistant *Staphylococcus aureus* (MRSA), vancomycin-resistant *Enterococcus* (VRE), *Streptococcus pneumoniae*, *Mycobacterium tuberculosis*, *Clostridioides difficile*, and *Bacillus anthracis*, with minimum inhibitory concentration values ranging from 0.005 to 0.5 μg mL^−1^.^1^ Teixobactin is especially promising as a drug candidate, because bacteria do not appear to be able to develop appreciable resistance to the antibiotic.^4^ With the rising threat of antibiotic resistance juxtaposed with a diminishing pipeline of new antibiotics, teixobactin offers a new hope in combating these drug-resistant pathogens.^5^

Initial reports established that the mode of action of teixobactin involves disrupting cell wall biosynthesis and ultimately causing cell lysis by binding to the peptidoglycan building block lipid II, the wall teichoic acid precursor lipid III, and related cell wall precursors.^1,6^ Teixobactin binds to the pyrophosphate groups of lipids II and III and related undecaprenyl-pyrophosphate containing immutable cell wall precursors, making it challenging for bacteria to acquire resistance to teixobactin. X-ray crystallographic studies of teixobactin analogues subsequently suggested a working model for the antibiotic action of teixobactin in which teixobactin binds to these cell wall precursors as dimers, higher-order assemblies, or fibrils formed through antiparallel β-sheet interactions.^7,8^ This model of antiparallel β-sheet assembly was corroborated by the recent report of a solid-state NMR structure of a teixobactin analogue complexed with lipid II in membranes, in which the teixobactin analogue assembles into antiparallel β-sheets with its macrolactone ring coordinating to the pyrophosphates of lipid II.^9^ Cryogenic transmission electron microscopy (CryoEM) studies have permitted visualization of the sheet-like assemblies formed by teixobactin and concurrent cell wall damage to *B. subtilis*.^10^ These structural studies, along with structure–activity relationship studies,^11^ demonstrate that the supramolecular assembly of teixobactin is important for its mechanism of action.

Fluorescence microscopy studies of teixobactin have allowed us to visualize the interactions between teixobactin and Gram-positive bacteria, and thus further elucidate the mechanism of action of teixobactin. Our laboratory recently characterized the cellular localization of a fluorescent teixobactin analogue in a variety of Gram-positive bacteria, and found that the antibiotic binds to the septa and sidewalls of the bacteria and forms large aggregates.^12^ Optical imaging by Weingarth *et al.* demonstrated the Arg_4_,Leu_10_-teixobactin analogue forms micron-sized patches in giant unilamellar vesicles (GUVs) doped with fluorescent lipid II, suggesting that teixobactin also disrupts cell wall biosynthesis by sequestering lipid II in clusters.^9^

Collectively, these structural and optical studies have demonstrated that teixobactin is a supramolecular antibiotic, in which supramolecular assembly is integral to its mechanism of action. The studies have also illustrated that the mechanism of action of teixobactin—and particularly the cellular and downstream effects triggered by binding to lipid II and related cell wall precursors—are more complex than initially understood.

In the current study, we set out to use fluorescence microscopy to further probe the mechanism of action of teixobactin and to provide additional insights into its supramolecular assembly on bacteria. Fluorescence microscopy alone does not provide sufficient spatial resolution to discern the proximity of individual molecules at the nanometer scale. We thus envisioned using FRET microscopy—with teixobactin analogues bearing two different FRET partners—to identify co-localization of individual molecules within a few nanometers of each other on the surface of bacteria. The synthesis of fluorescent teixobactin analogues that we had previously reported only enabled the preparation of teixobactin analogues bearing a sulforhodamine B label; other fluorophores were not compatible with this synthetic method.^12^ Here we describe a versatile synthesis that enables the preparation of teixobactin analogues bearing a variety of fluorophores. We then use these labeled teixobactin analogues to better understand how teixobactin interacts with and acts upon bacteria, through structured illumination microscopy, FRET microscopy, and time-dependent microscopy studies with *B. subtilis*.

## Results and discussion

### Labeling of teixobactin analogues

**Fig. 1:**
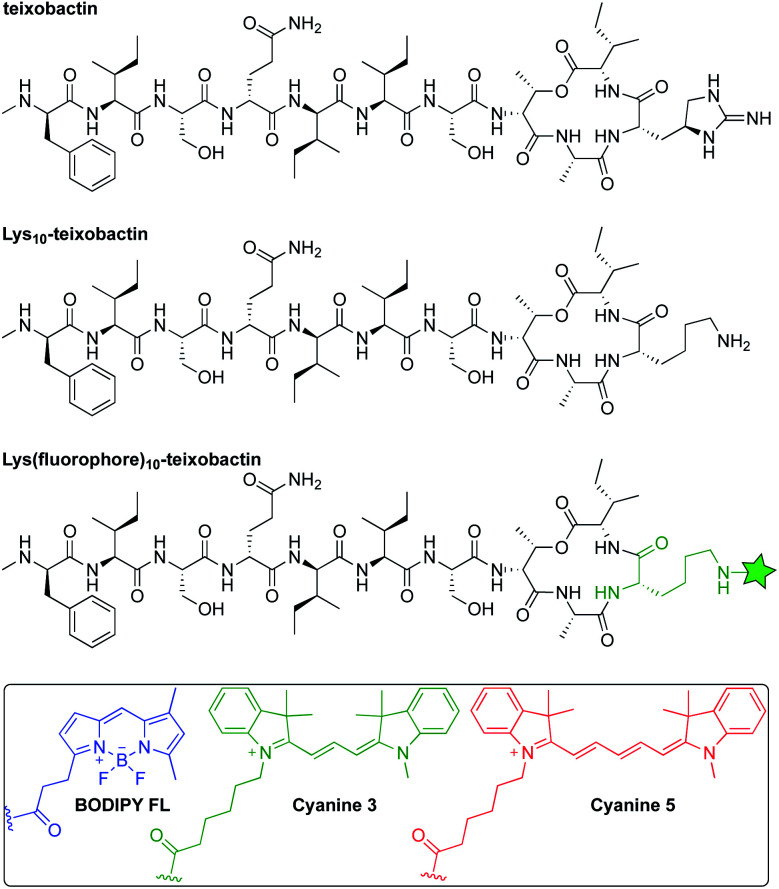
Structures of teixobactin, Lys_10_-teixobactin, and Lys(fluorophore)_10_-teixobactin, where the green star represents an amide conjugate of a fluorophore.

Inspired by reports that teixobactin tolerates substitution at position 10,^11–15^ we developed a strategy to allow the facile preparation of active teixobactin analogues bearing different fluorophores at position 10. Starting with Lys_10_-teixobactin, we found that treatment with fluorophore NHS esters cleanly gave reaction at the Lys_10_ side chain, without reaction at the N-terminal amino group of the *N*-methyl-d-phenylalanine. Using this reaction (Scheme S1†), we prepared derivatives bearing BODIPY FL, Cy3, and Cy5 fluorophores (Fig. 1). We compared these Lys(fluorophore)_10_-teixobactin derivatives to the corresponding homologues containing arginine at position 10 and the fluorophore at position 9, Lys(fluorophore)_9_,Arg_10_-teixobactin, as position 9 can also accommodate fluorophore labeling without abrogation of antibiotic activity.

The synthesis of both sets of compounds proceeded smoothly (Scheme S1†). Treatment of either Lys_10_-teixobactin or Lys_9_,Arg_10_-teixobactin with 1.2 equivalents of the NHS esters of the corresponding fluorophore in DMF in the presence of diisopropylethylamine for 10–60 minutes, followed by purification by reverse-phase HPLC, afforded the corresponding labeled teixobactin analogue. Use of 5 mg (3.4 μmol) of peptide typically yielded 1–3 mg of Lys(BDY FL)_10_-teixobactin, Lys(Cy3)_10_-teixobactin, or Lys(Cy5)_10_-teixobactin, or the corresponding Lys(fluorophore)_9_,Arg_10_-teixobactin analogues, as the trifluoroacetate (TFA) salts, which was sufficient for all subsequent studies.

### Antibiotic activity of fluorescent teixobactin analogues

Minimum inhibitory concentration (MIC) assays established that the teixobactin analogues bearing fluorophores at position 10 generally exhibit superior antibiotic activity to the corresponding Arg_10_-teixobactin analogues bearing fluorophores at position 9. Table 1 summarizes the activities of these analogues against the Gram-positive bacteria *Bacillus subtilis* and *Staphylococcus epidermidis*. We performed these assays in the absence or presence of 0.002% polysorbate 80, which we and others have found to improve the antibiotic activity of teixobactin and teixobactin analogues in MIC assays.^1,8,12^ Controls with *Escherichia coli* confirm that these analogues are selective for Gram-positive bacteria and establish that the fluorophores do not impart non-specific antibiotic activity against Gram-negative bacteria. Collectively, these experiments show that Lys_10_-teixobactin and Lys_9_,Arg_10_-teixobactin can tolerate a range of fluorophores that differ in size, charge, and hydrophobicity without causing complete loss of antibiotic activity, and that a positively charged residue, such as l-*allo*-enduracididine or arginine, is not required at position 10 for antibiotic activity.^15^

**Table 1:** MIC values of teixobactin analogues in μg mL^−1^ with 0% and 0.002% polysorbate 80

	*Bacillus subtilis* ATCC 6051	*Staphylococcus epidermidis* ATCC 14990	*Escherichia coli* ATCC 10798
Lys(BDY FL)_10_-teixobactin	2^a^	4^a^	>32^a^
	0.125^b^	1^b^	>32^b^
Lys(BDY FL)_9_,Arg_10_-teixobactin	8^a^	8^a^	>32^a^
	2^b^	2^b^	>32^b^
Lys(Cy3)_10_-teixobactin	4^a^	4^a^	>32^a^
	1^b^	2^b^	>32^b^
Lys(Cy3)_9_,Arg_10_-teixobactin	4^a^	8^a^	>32^a^
	2^b^	8^b^	>32^b^
Lys(Cy5)_10_-teixobactin	8^a^	16^a^	>32^a^
	2^b^	16^b^	>32^b^
Lys(Cy5)_9_,Arg_10_-teixobactin	8^a^	16^a^	>32^a^
	4^b^	8^b^	>32^b^
Arg_10_-teixobactin	1^a^	2^a^	>32^a^
	<0.03^b^	0.5^b^	>32^b^
Lys_10_-teixobactin	1^a^	1–2^a^	>32^a^
	<0.03^b^	0.5^b^	>32^b^

a Culture media containing 0% polysorbate 80.

b Culture media containing 0.002% polysorbate 80.

**Fig. 2:**
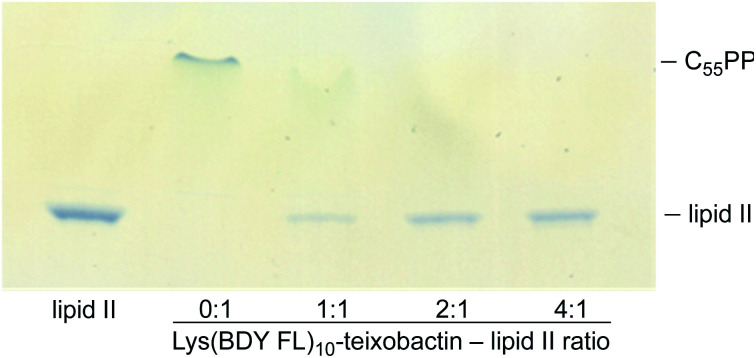
Effect of Lys(BDY FL)_10_-teixobactin on PBP2-catalyzed conversion of lipid II and release of undecaprenyl pyrophosphate (C_55_PP) in an *in vitro* transglycosylation assay. Reaction mixtures were analyzed by thin-layer chromatography with phosphomolybdic acid staining. Lipid II was run in lane 1 as a control.

An *in vitro* transglycosylation assay establishes that labeling at position 10 does not abrogate interaction with lipid II. In this assay, lipid II is incubated with *S. aureus* penicillin binding protein 2 (PBP2), which catalyzes the polymerization of lipid II and results in the release of undecaprenyl pyrophosphate (C_55_PP). The lipid II and C_55_PP are then detected by thin-layer chromatography with phosphomolybdic acid staining. Previous studies have shown that addition of teixobactin inhibits lipid II transglycosylation, and thus the release of C_55_PP, in a concentration-dependent fashion.^1^ When we treated lipid II with various stoichiometries of Lys(BDY FL)_10_-teixobactin in a similar fashion, we also observed inhibition of C_55_PP release in a concentration-dependent fashion (Fig. 2), indicating that Lys(BDY FL)_10_-teixobactin interferes with the PBP2-catalyzed reaction by binding to lipid II. When lipid II was incubated only with PBP2, without Lys(BDY FL)_10_-teixobactin, lipid II was completely polymerized and a strong C_55_PP band was observed. A 1 : 1 molar ratio of Lys(BDY FL)_10_-teixobactin and lipid II resulted in substantial reduction in C_55_PP release and concurrent increased appearance of unconverted lipid II. A 2 : 1 molar ratio resulted in an almost complete inhibition of the PBP2-catalyzed reaction. The lipid II band intensity only marginally increased at a 4 : 1 molar ratio, suggesting that transglycosylation is almost completely inhibited at 2 : 1, as observed with native teixobactin.^1^ Collectively, the MIC and transglycosylation assays show that position 10 provides a suitable site for labeling teixobactin that does not affect overall target interaction.

### Structured illumination microscopy of fluorescent teixobactin analogues in *B. subtilis*

**Fig. 3:**
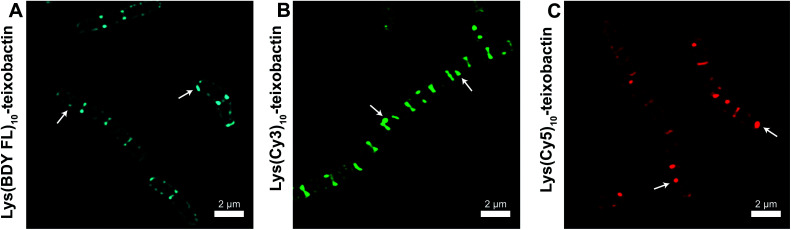
Representative SIM micrographs of *B. subtilis* stained with (A) 1 μg mL^−1^ Lys(BDY FL)_10_-teixobactin, (B) Lys(Cy3)_10_-teixobactin, (C) and Lys(Cy5)_10_-teixobactin. Scale bars are 2 μm. White arrows indicate bright patches of the fluorescent teixobactin analogue. See Fig. S1† for expansions of each micrograph.

We evaluated the staining of the fluorescent teixobactin analogues in *B. subtilis* using structured illumination microscopy (SIM) to visualize interactions between the fluorescent antibiotics and the molecular targets in *B. subtilis*. SIM is a super-resolution microscopy technique that can provide roughly twice the resolution of conventional confocal microscopy, thus enabling the study of the interactions of teixobactin with micron-sized bacteria at resolution approaching 100 nm. We treated *B. subtilis* with 1 μg mL^−1^ of Lys(BDY FL)_10_-teixobactin, Lys(Cy3)_10_-teixobactin, or Lys(Cy5)_10_-teixobactin in sodium phosphate buffer, washed the bacteria, and then imaged by SIM microscopy (Fig. 3). We chose 1 μg mL^−1^, because it results in bright staining and is below the MIC values of these fluorescent teixobactin analogues against *B. subtilis*. Micrographs of *B. subtilis* stained with Lys(BDY FL)_10_-teixobactin, Lys(Cy3)_10_-teixobactin, and Lys(Cy5)_10_-teixobactin revealed staining of the septa and sidewalls of the bacteria (Fig. 3A–C). Labeling of division sites and sidewalls is consistent with the antibiotic targeting lipid II and related undecaprenyl-coupled cell wall precursors associated with cell wall biosynthesis and cell division.^16,17^

The higher resolution provided by SIM enables clear observation of small features that are difficult to observe by traditional confocal microscopy. A few bright patches of fluorescent teixobactin are apparent on the sidewalls and poles of a number of the bacteria that do not appear to be associated with the septa and are brighter than the typical staining of the sidewalls (see white arrows in Fig. 3). These bright patches suggest clustering of pyrophosphate-containing cell wall precursors induced by aggregation of the teixobactin analogues, in a fashion consistent with its supramolecular mechanism of action. Such clustering has also been observed when GUVs doped with fluorescent lipid II were treated with a teixobactin analogue,^9^ and in Gram-positive bacteria treated with fluorescent analogues of daptomycin^18–20^ and nisin.^21,22^

### FRET studies of fluorescent teixobactin analogues in *B. subtilis*

**Fig. 4:**
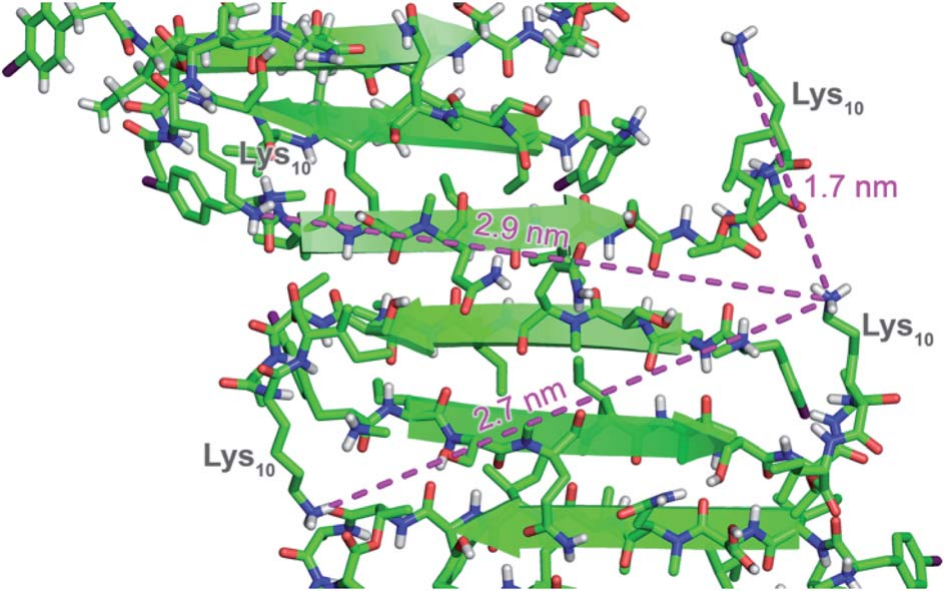
X-ray crystallographic structure of a derivative of Lys_10_-teixobactin illustrating proximity between nearby Lys_10_ residues (PDB 6E00).^7^

To further understand the self-assembly of teixobactin on bacteria at resolution higher than is possible by SIM microscopy, we turned to FRET microscopy. For these studies, we used fluorescent analogues of teixobactin bearing the FRET partners Cy3 and Cy5 and sought to detect intimate proximity (<10 nm) between the labeled teixobactin molecules. In crystallographic studies of teixobactin analogues, we have observed that the teixobactin molecules assemble into dimers and higher-order assemblies through β-sheet formation (Fig. 4).^7,8^ In the supramolecular assemblies, the separation between Lys_10_ of nearby teixobactin molecules is 2–3 nm, which is well under the Förster radius for the Cy3–Cy5 FRET pair (5–6 nm).^23–26^ We thus stained *B. subtilis* with both Lys(Cy3)_10_-teixobactin and Lys(Cy5)_10_-teixobactin, imaged the bacteria at a wavelength selective for Cy3 (490 nm), and observed at wavelengths selective for Cy5 (660–700 nm) to identify FRET emission associated with intermolecular β-sheet formation.

**Fig. 5:**
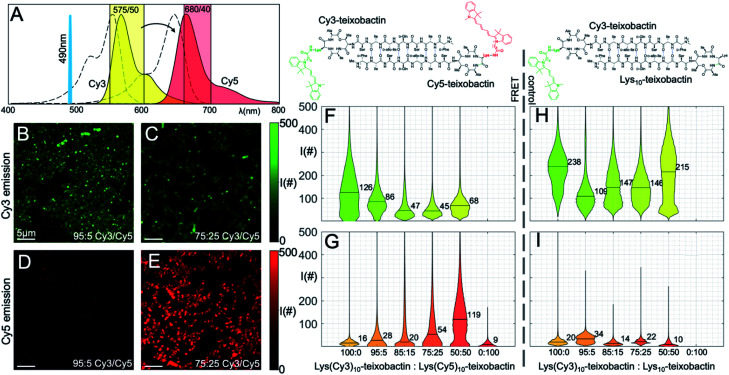
FRET microscopy with Lys(Cy3)_10_-teixobactin and Lys(Cy5)_10_-teixobactin in *B. subtilis*. (A) Fluorescence emission spectra of the Cy3 and Cy5 fluorophores are shown (colored in yellow and red, respectively) together with the absorption spectra (dashed lines), the excitation laser line at 490 nm, and the two spectral bands used to collect the fluorescence (colored shaded rectangles). (B and C) Representative micrographs taken in the Cy3 spectral band at 95 : 5 and 75 : 25 Cy3 to Cy5 ratio (scale bars are 5 μm). (D and E) Representative micrographs taken in the Cy5 spectral emission band at 95 : 5 and 75 : 25 Cy3 : Cy5 ratio (scale bars are 5 μm). (F and G) Distribution of photon counts per pixel (*I*#) as a function of each concentration for all micrographs in the two spectral bands. Median values are reported numerically on each violin plot. (H and I) Control experiments performed with decreasing ratios of Lys(Cy3)_10_-teixobactin and unlabeled Lys_10_-teixobactin. See Fig. S2 and S3† for additional representative micrographs.

We treated *B. subtilis* with mixtures of Lys(Cy3)_10_-teixobactin and Lys(Cy5)_10_-teixobactin, in 100 : 0, 95 : 5, 85 : 15, 75 : 25, 50 : 50, and 0 : 100 ratios, with a net concentration of 1 μg mL^−1^. We then imaged the samples on a scanning confocal microscope by selectively illuminating Lys(Cy3)_10_-teixobactin using 490 nm excitation. Fluorescence micrographs were collected in the emission bands of Cy3 and Cy5, and the emission from each fluorophore was compared by plotting the distribution of the photon counts obtained from each sample (Fig. 5). As the fraction of Lys(Cy5)_10_-teixobactin increased, the emission of Cy5 increased, and the ratio of the photon counts from the Cy3 and Cy5 channels decreased (Fig. 5F and G), providing compelling evidence for FRET between teixobactin molecules bound to *B. subtilis*.

The violin plots depict the distributions of photon counts across all images for each sample. Some of these distributions are bimodal, with a low intensity population due to the background with low photon counts (*i.e.*, regions where no bacteria are present), and another population of higher intensity accounting for the actual fluorescence emission. In the more crowded samples, in which the bacteria are covering the entire field of view and there is no background pixel population, the pixel intensity distribution is unimodal. Representative micrographs recorded for the 95 : 5 and 75 : 25 ratios (Fig. 5B–E) illustrate the emission from the Cy3 and Cy5 channels and provide additional evidence for FRET.

As a control experiment, we treated *B. subtilis* with mixtures of Lys(Cy3)_10_-teixobactin and Lys_10_-teixobactin (unlabeled), in 100 : 0, 95 : 5, 85 : 15, 75 : 25, 50 : 50, and 0 : 100 ratios, with a net concentration of 1 μg mL^−1^. Upon imaging these samples by excitation at 490 nm, we observed strong emission in the Cy3 channel and negligible emission in the Cy5 channel (Fig. 5H–I). This observation provides further evidence that the Cy5 emission observed in the mixtures of Lys(Cy3)_10_-teixobactin and Lys(Cy5)_10_-teixobactin in Fig. 5 results from FRET and not from the tail of Cy3 emission above 660 nm. Interestingly, the dilution of Lys(Cy3)_10_-teixobactin with Lys_10_-teixobactin does not result in a ratio-dependent diminution of Cy3 emission (Fig. 5H). Instead, the Cy3 emission from the 50 : 50 mixture of Lys(Cy3)_10_-teixobactin and Lys_10_-teixobactin is comparable to that of the Cy3 emission from the 100 : 0 mixture and is greater than that of the 95 : 5, 85 : 15, and 75 : 25 mixtures. The relative brightness of Cy3 emission from the 50 : 50 mixture may reflect reduced fluorescence quenching associated upon dilution of the fluorophore within the assemblies on the bacteria. Similar effects have been noted when 50 : 50 mixtures of various labeled and unlabeled antibiotics have been used to stain bacteria, including mixtures of labeled and unlabeled teixobactin,^12^ vancomycin^17^ and daptomycin.^20^

The FRET between Lys(Cy3)_10_-teixobactin and Lys(Cy5)_10_-teixobactin indicates that the teixobactin molecules are intimately co-localizing (<10 nm) on *B. subtilis* and is consistent with the model put forth in Fig. 4, in which teixobactin forms dimers and higher order assemblies on bacteria. This FRET experiment is significant, because such close proximity (<10 nm) between individual molecules cannot be discerned by confocal microscopy or even super-resolution microscopy. Although it is not possible to dissect the details of the molecular assemblies or their interactions with the bacteria or the cell wall precursors, the observation of FRET provides further evidence that teixobactin binds to the pyrophosphate groups of lipid II and related cell wall precursors as dimers or higher-order assemblies.^1,7–9^^,^‡‡FRET studies have also been used to establish the oligomerization of daptomycin on membranes. For examples, see: (A) J. K. Muraih, A. Pearson, J. Silverman and M. Palmer, *Biochim. Biophys. Acta*, 2011, **1808**, 1154–1160. (B) J. K. Muraih and M. Palmer, *BBA, Biochim. Biophys. Acta, Biomembr.*, 2012, **1818**, 1642–1647.

### Time-dependent binding and bactericidal activity in *B. subtilis*

Teixobactin has been reported to be bactericidal and bacteriolytic at concentrations above its minimum inhibitory concentration.^1,6^ To visualize the binding of teixobactin to bacteria and bacteriolysis over time, we incubated *B. subtilis* with Lys(BDY FL)_10_-teixobactin and propidium iodide (PI) and analyzed their interactions by fluorescence microscopy. Propidium iodide is widely used as a fluorescent reporter of cell viability and localizes in the DNA of mammalian and bacterial cells in which the integrity of the cell membrane has been significantly compromised. In these experiments, we used a 50 : 50 mixture of Lys(BDY FL)_10_-teixobactin and Lys_10_-teixobactin, at 3 μg mL^−1^ final concentration of each, to ensure bright staining and bactericidal concentrations of the antibiotic. *B. subtilis* was incubated with the teixobactin analogues, and aliquots were removed at varying time points (0.5, 30, 60, 90, and 120 min). After each aliquot was removed, the bacteria were immediately washed, stained with PI, washed again, and imaged by fluorescence microscopy.

**Fig. 6:**
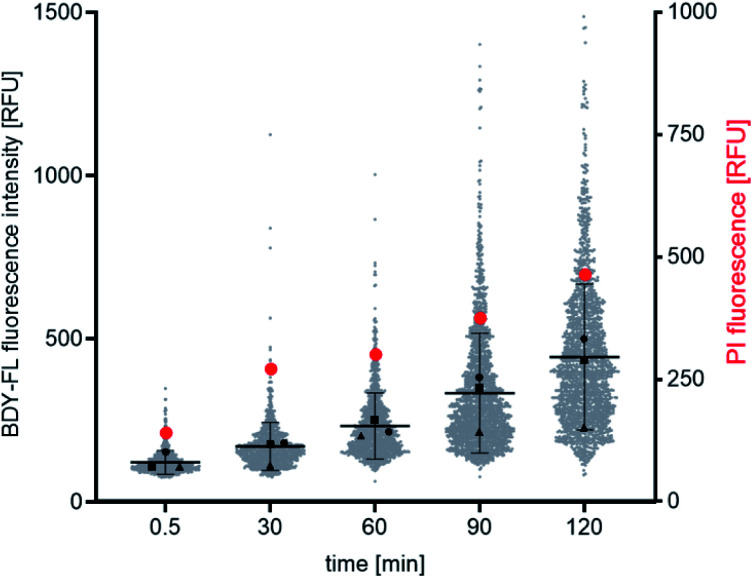
Time-dependent fluorescence microscopic binding studies with *B. subtilis*. Distribution of Lys(BDY FL)_10_-teixobactin and PI fluorescence intensities as shown as a function of time. Gray dots represent the mean fluorescence intensity of Lys(BDY FL)_10_-teixobactin measured in single cells in three independent experiments. The mean ± SD is indicated by black lines. Mean values of the individual experiments are indicated by black triangles, squares, and circles. Red circles represent the mean fluorescence intensity of PI measured in single cells in three independent experiments.

Both the Lys(BDY FL)_10_-teixobactin fluorescence and the PI fluorescence increased over time, indicating that the proportion of bacteria with severe membrane damage correlates with the amount of Lys(BDY FL)_10_-teixobactin bound to *B. subtilis* (Fig. 6). This trend was also observed when the PI fluorescence was analyzed as a function of the Lys(BDY FL)_10_-teixobactin fluorescence in individual bacteria, with a shift to higher fluorescence intensities in both channels becoming apparent with increasing incubation times (Fig. S4,† gray box). At longer incubation times (≥90 min), a subset of cells characterized by a high fluorescence intensity in the BDY FL channel (2.6% at 90 min, 7.2% at 120 min) lacked comparable fluorescence in the PI channel (Fig. S4,† yellow box). Examination of the individual micrographs provides insights into this observation.

**Fig. 7:**
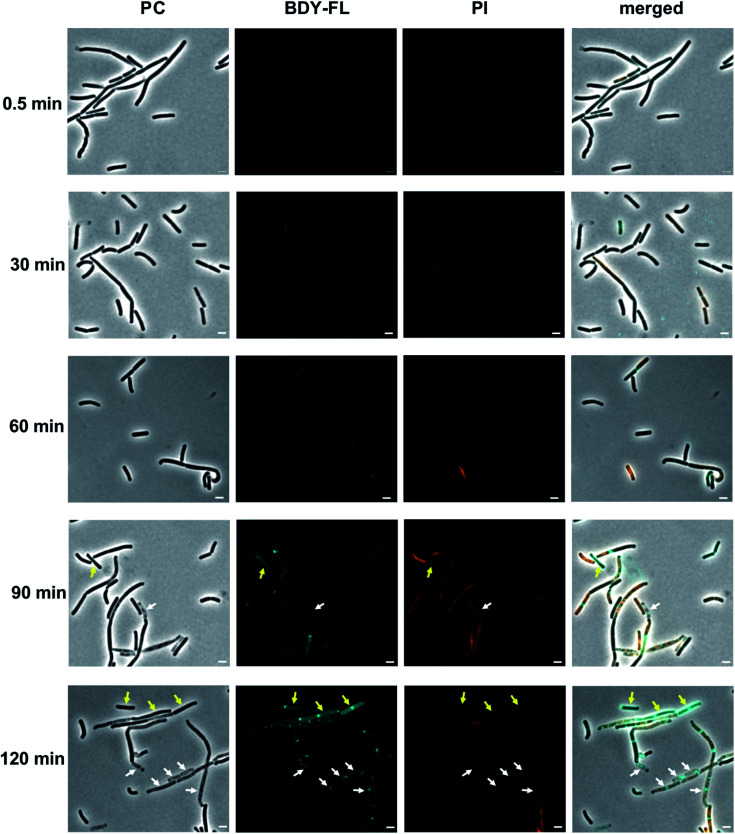
Interaction of Lys(BDY FL)_10_-teixobactin (BDY FL) and propidium iodide (PI) with *B. subtilis* over time. Representative micrographs taken during the time-dependent binding studies. Phase contrast (PC), BDY FL, PI, and merged channels are shown. Scale bars are 2 μm. Micrographs were acquired and processed using the same settings, to allow comparisons in intensity over time. Arrows highlight bacteria with a high BDY FL signal but without PI signal. White arrows highlight bacteria that are translucent in PC, and yellow arrows highlight bacteria with a regular appearance in PC.

In the fluorescence micrographs, binding of Lys(BDY FL)_10_-teixobactin to the septa and sidewalls of the *B. subtilis* is observed, with an intensity that increases with increasing incubation times (Fig. 7). Inspection of the individual bacteria revealed that some are, indeed, dead bacteria that have lysed and have lost their cytosolic content, including their DNA, as indicated by their translucent appearance in the phase contrast image (Fig. 7, white arrows). These lysed bacteria are not stained by propidium iodide due to the loss of their DNA. Another subset of the bacteria accumulated Lys(BDY FL)_10_-teixobactin over the entire bacterium with a strong septal or polar focus. These bacteria, however, did not show any sign of membrane damage or death (Fig. 7, yellow arrows). The differences between the subsets of these cells might result from being at different stages in the division cycle when treated with Lys(BDY FL)_10_-teixobactin. In addition, the apparent non-susceptibility of these bacteria to membrane damage by the accumulation of bound Lys(BDY FL)_10_-teixobactin might result from upregulation of cell wall biosynthesis that is coupled with increased precursor pool levels. In this population of cells, balanced elevation of precursor synthesis and turnover may allow these cells to maintain cell wall biosynthesis and integrity to counteract Lys(BDY FL)_10_-teixobactin action up to a critical limit (with regard to time and concentration). The fate of these bacteria might be unraveled by future live-cell microscopy experiments.

## Conclusions

Selective labeling of teixobactin analogues containing lysine with NHS esters permits the facile preparation of fluorescent teixobactin analogues bearing a variety of different fluorophores. Labeling at either position 9 or position 10 yields fluorescent teixobactin analogues with antibiotic activity, with Lys(fluorophore)_10_-teixobactin derivatives generally having superior antibiotic activity to Lys(fluorophore)_9_Arg_10_-teixobactin derivatives. Structured-illumination microscopy reveals that Lys(BDY FL)_10_-teixobactin, Lys(Cy3)_10_-teixobactin, and Lys(Cy5)_10_-teixobactin analogues bind the septa and sidewalls of *B. subtilis*. Additional bright patches of the fluorescent teixobactin analogues on the surface of the bacteria are also observed, reflecting clustering of the antibiotics with peptidoglycan precursors.

FRET microscopy using Lys(Cy3)_10_-teixobactin and Lys(Cy5)_10_-teixobactin reveals intimate co-localization of teixobactin molecules on *B. subtilis* in a fashion consistent with dimers or higher-order assemblies binding to cell wall precursors. To our knowledge, this study constitutes the first use of FRET microscopy with antibiotics bearing FRET partners to elucidate the molecular details of the interactions of the antibiotics with bacteria. Studies of the interaction of Lys(BDY FL)_10_-teixobactin with *B. subtilis* over time while monitoring membrane integrity reveal concurrent binding of teixobactin and loss of membrane integrity in some of the cells, but binding with retention of membrane integrity in others. Although fluorophores can perturb the interactions of small peptides with their molecular targets,^27^ we anticipate that the ready access to fluorescent teixobactin analogues described herein will enable many additional studies that further the understanding of the unusual mechanism of action of teixobactin to help unlock its promise as a new antibiotic.

## Data availability

Additional data can be found in the ESI† document.

## Author contributions

M. A. M. and J. S. N. designed the project. M. A. M., C. R. J., B. T. N., M. H. H., and M. M. synthesized and purified teixobactin analogues. M. A. M. performed structured illumination microscopy experiments. M. A. M. and A. V. carried out FRET microscopy experiments, and A. V. performed FRET quantitative analysis. F. G. performed and analyzed time-lapse microscopy experiments. T. S. and M. A. performed *in vitro* transglycosylation assays. M. A. M. performed MIC assays. The manuscript was written with contributions from M. A. M., J. S. N., A. V., F. G., T. S.

## Conflicts of interest

J. S. N. and the University of California, Irvine are the recipients of a subcontract from NovoBiotic Pharmaceuticals, LLC.

## Supplementary Material

SC-013-D2SC01388F-s001
