# Supplementary material for: Visualizing the mode of action and supramolecular assembly of teixobactin analogues in Bacillus subtilis
Source: Chem Sci. 2022 May 13;13(26):7747–54. doi: 10.1039/d2sc01388f (PMC9258396; doi:10.1039/d2sc01388f)
Supplement: SC-013-D2SC01388F-s001 [file SC-013-D2SC01388F-s001.pdf]

Electronic Supplementary Material (ESI) for Chemical Science.
This journal is © The Royal Society of Chemistry 2022

Supporting Information for:

Visualizing the Mode of Action and Supramolecular Assembly of Teixobactin Analogues in *Bacillus subtilis*

Michael A. Morris,[a] Alexander Vallmitjana,[b] Fabian Grein,[c,d] Tanja Schneider,[c] Melina Arts,[c] Chelsea R. Jones,[a] Betty T. Nguyen,[a] Mohammad H. Hashemian,[a] Melody Malek,[a] Enrico Gratton,[b] and James S. Nowick*,[a,e]

[a]Department of Chemistry, University of California, Irvine, Irvine, California 92697, United States

[b]Laboratory for Fluorescence Dynamics, Department of Biomedical Engineering, University of California, Irvine, Irvine, California 92697, United States

[c]Institute for Pharmaceutical Microbiology, University of Bonn, University Clinic Bonn, Bonn 53115, Germany

[d]German Center for Infection Research (DZIF), partner site Bonn-Cologne, Bonn 53115, Germany

[e]Department of Pharmaceutical Sciences, University of California, Irvine, Irvine, California 92697, United States

E-mail: jsnowick@uci.edu

Table of Contents

Materials and Methods S4

Materials S4

Methods for Synthesis, Purification, and Analysis of Peptides S4

Synthesis of Lys_{10}-teixobactin S5

Synthesis of Lys_9,Arg_{10}-teixobactin S5

Representative Procedure for Labeling Lys_{10}-teixobactin and Lys_9,Arg_{10}-teixobactin with Fluorophore NHS Esters: Synthesis of Lys(BDY FL)$_{10}$-teixobactin S5

Scheme S1. Regioselective Labeling of Lys_{10}- and Lys_9,Arg_{10}-teixobactin using Fluorophore NHS Esters S6

Table S1. Yield and Mass Spectrometric Data of Purified Fluorescent Teixobactin Analogues S6

Confirmation of Regioselective Labeling of Lys_{10} in Lys(BDY FL)$_{10}$-teixobactin S7

Preparation of DMSO Stock Solutions S11

MIC Assays S11

In Vitro PBP2 Transglycosylation Assay using Lipid II S11

Fluorescence Microscopy Studies S11

Table S2. Volumes of Lys(Cy3)$_{10}$-teixobactin and Lys(Cy5)$_{10}$-teixobactin Stock Solutions used to Prepare FRET Samples S14

Table S3. Volumes of Lys(Cy3)$_{10}$-teixobactin and Lys_{10}-teixobactin Stock Solutions used to Prepare FRET Samples S14

References for Supporting Information S15

Supplementary data S17

Figure S1. Micrograph Expansions from Figure 3 S17

Figure S2. Additional Representative Micrographs from the Lys(Cy3)$_{10}$-teixobactin/Lys(Cy5)$_{10}$-teixobactin FRET Experiments S20

Figure S3. Additional Representative Micrographs from the Lys(Cy3)$_{10}$-teixobactin/Lys_{10}-teixobactin FRET Control Experiments S21

Figure S4. Analysis of the PI Fluorescence Distribution as a Function of Lys(BDY FL)$_{10}$-teixobactin Fluorescence in Individual *B. subtilis* Cells at Different Time Points S22

Characterization of teixobactin analogues S23

Lys_{10}-teixobactin S23
HPLC Trace and MALDI Mass Spectrum

Lys(BDY FL)$_{10}$-teixobactin S26
HPLC trace and ESI Mass Spectrum

Lys(Cy3)$_{10}$-teixobactin S30
HPLC Trace and MALDI Mass Spectrum

Lys(Cy5)$_{10}$-teixobactin S35
HPLC Trace and MALDI Mass Spectrum

Lys$_{9}$,Arg$_{10}$-teixobactin S40
HPLC Trace and MALDI Mass Spectrum

Lys(BDY FL)$_{9}$,Arg$_{10}$-teixobactin S43
HPLC trace and ESI Mass Spectrum

Lys(Cy3)$_{9}$,Arg$_{10}$-teixobactin S45
HPLC Trace and MALDI Mass Spectrum

Lys(Cy5)$_{9}$,Arg$_{10}$-teixobactin S48
HPLC Trace and MALDI Mass Spectrum

Materials and Methods

Materials. Amino acids, coupling agents, 2-chlorotrityl chloride resin, DIC, and triisopropylsilane were purchased from Chem-Impex. DMF (amine-free), DIPEA, 2,4,6-collidine, and piperidine were purchased from Alfa-Aesar. DMAP and polysorbate 80 were purchased from Acros Organics. BODIPY FL NHS ester, Cy3 NHS ester, and Cy5 NHS ester were purchased from Lumiprobe. HPLC-grade acetonitrile, and dichloromethane were purchased from Fisher Scientific. TFA and hexafluoroisopropanol were purchased from Oakwood Chemical. Reagent-grade solvents, chemicals, amino acids, and resin were used as received, with the exception of dichloromethane, which was dried through an alumina column under argon, and dimethylformamide, which was dried through an alumina column and an amine scavenger resin column under argon.

Methods for Synthesis, Purification, and Analysis of Peptides. Solid-phase peptide synthesis was carried out manually in a solid phase reaction vessel.[1] Analytical reverse-phase HPLC was performed on an Agilent 1200 instrument equipped with an Aeris PEPTIDE 2.6u XB-C18 column (Phenomonex). Preparative reverse-phase HPLC was performed on a Rainin Dynamax instrument equipped with a Zorbax SB-C18 column (Agilent) for unlabeled teixobactin analogues or a Zorbax 9.4 x 250 mm C18 semipreparative column (Agilent) for fluorescent teixobactin analogues. UV detection (214 nm) was used for analytical and preparative HPLC. HPLC grade acetonitrile and 18 MΩ deionized water, each containing 0.1% trifluoroacetic acid, were used for analytical and preparative reverse-phase HPLC. Matrix-assisted laser desorption ionization time-of-flight (MALDI-TOF) mass spectrometry was performed on an AB SCIEX TOF/TOF 5800 system and 2,5-dihydroxybenzoic acid was used as the sample matrix. Electrospray ionization (ESI) mass spectrometry was performed on an LCT ESI LC-TOF in

positive mode. High resolution mass spectrometry (HRMS) was performed on a Waters Synapt G2 in positive ion mode. All peptides were prepared and used as the trifluoroacetate salts and were assumed to have one trifluoroacetate ion per ammonium group present in each peptide.

Synthesis of Lys_{10}-teixobactin. Lys_{10}-teixobactin was prepared as the trifluoroacetate salt by solid-phase peptide synthesis followed by solution phase cyclization, as previously described.[2,3] A typical synthesis on a 0.1 mmol scale afforded 10–20 mg of Lys_{10}-teixobactin.

Synthesis of Lys_9,Arg_{10}-teixobactin. Lys_9,Arg_{10}-teixobactin was prepared as the trifluoroacetate salt in a similar fashion to Lys_{10}-teixobactin.[2,3]

Representative Procedure for Labeling Lys_{10}-teixobactin and Lys_9,Arg_{10}-teixobactin with Fluorophore NHS Esters: Synthesis of Lys(BDY FL)$_{10}$-teixobactin. 5 mg of BODIPY FL NHS ester was dissolved in 100 μL of dry DMSO to create a 50 mg/mL stock solution, which was wrapped in black felt to protect it from light and stored in a desiccator in a −20 °C freezer for subsequent reactions. 5.1 mg of Lys_{10}-teixobactin (3.5 μmol, 1.0 equiv) was weighed into a tared Eppendorf tube using an analytical balance. The peptide was dissolved in 59.3 μL of dry DMF and 3.1 μL of DIPEA (17.7 μmol, 5.0 equiv) was slowly added to the solution. The solution was vortexed to ensure homogeneity and then centrifuged to bring the solution to the bottom of the tube. To this solution, 37.6 μL of 50 mg/mL BODIPY FL NHS ester (4.2 μmol, 1.2 equiv) was added and the solution was mixed by pipetting (Scheme S1). The Eppendorf tube was covered with black felt and placed on a rocker. The reaction progress was monitored by ESI-MS and analytical HPLC (using 214 and 488 nm for detection) and the reaction was complete within 10–60 min. The reaction mixture was diluted with 3 mL of 40% (*v/v*) CH_3CN in water and was purified by reverse-phase HPLC with H_2O/CH_3CN (gradient elution of 20–50% CH_3CN with 0.1% TFA over 60 min) equipped with an Agilent Zorbax 9.4 x 250 mm C18 semipreparative column. The

C18 column was heated to 50 °C in a Sterlite plastic bin water bath equipped with a Kitchen Gizmo Sous Vide immersion circulator to maintain the solubility of the peptide during purification. Pure fractions were characterized by analytical HPLC and ESI-MS and subsequently collected, concentrated, and lyophilized. After lyophilization, 3.3 mg of Lys(BDY FL)$_{10}$-teixobactin (58% yield) was obtained as an orange powder.

Scheme S1. Regioselective labeling of Lys$_{10}$-teixobactin and Lys$_9$,Arg$_{10}$-teixobactin using fluorophore NHS esters.

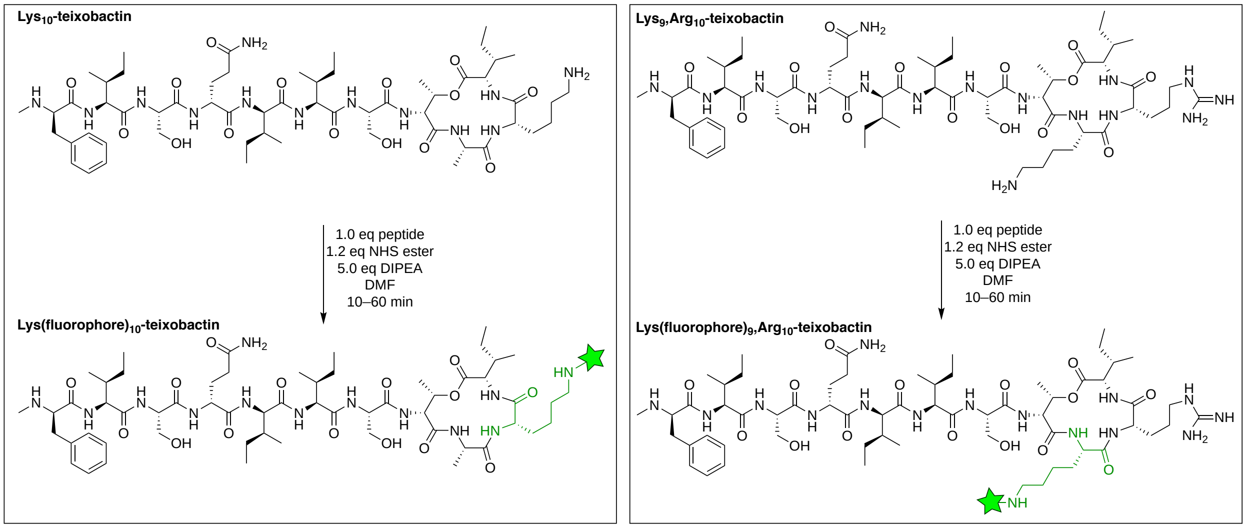

Lys(Cy3)$_{10}$-teixobactin and Lys(Cy5)$_{10}$-teixobactin were prepared using the same procedure. Lys(BDY FL)$_9$,Arg$_{10}$-teixobactin, Lys(Cy3)$_9$,Arg$_{10}$-teixobactin, Lys(Cy5)$_9$,Arg$_{10}$-teixobactin were prepared using the same procedure, except Lys$_9$,Arg$_{10}$-teixobactin was used instead of Lys$_{10}$-teixobactin. The yields of each purified fluorescent peptide are reported in Table S1, along with their corresponding mass spectrometric data.

Table S1. Yield and mass spectrometric data of purified fluorescent teixobactin analogues.

Fluorescent Analogue	% Yield	Calcd. MW as TFA salt	Calcd. *m/z* (LRMS)	Found *m/z* (LRMS)	Calcd. *m/z* (HRMS)	Found *m/z* (HRMS)
Lys(BDY FL)$_{10}$-teixobactin	58%	1604.59 (•1 TFA)	1490.8 [M+H]$^+$ (for ^{11}B isotopologue)	1491.1	735.9200 [M-F+H]$^{2+}$	735.9200
Lys(BDY FL)$_9$,Arg$_{10}$-teixobactin	54%	1803.72 (•2 TFA)	1575.9 [M+H]$^+$ (for ^{11}B isotopologue)	1575.8	N/A	N/A
Lys(Cy3)$_{10}$-teixobactin	53%	1883.15 (•2 TFA)	1655.0 [M]$^+$	1655.0	828.0022 [M+H]$^{2+}$	827.9991
Lys(Cy3)$_9$,Arg$_{10}$-teixobactin	43%	2082.28 (•3 TFA)	1740.1 [M]$^+$	1740.4	N/A	N/A
Lys(Cy5)$_{10}$-teixobactin	54%	1909.19 (•2 TFA)	1681.0 [M]$^+$	1681.1	841.0100 [M+H]$^{2+}$	841.0066
Lys(Cy5)$_9$,Arg$_{10}$-teixobactin	53%	2108.32 (•3 TFA)	1766.1 [M]$^+$	1766.3	N/A	N/A

Confirmation of Regioselective Labeling of Lys_{10} in Lys(BDY FL)$_{10}$-teixobactin. ^{1}H NMR spectroscopy and TOCSY NMR spectroscopy on a 3.3 mM solution of Lys(BDY FL)$_{10}$-teixobactin in CD_3SOCD_3 established that labeling occurred at the ε-position of Lys_{10}. A pair of distinct resonances are observed for the diastereotopic protons of the ammonium group *N*-Me-D-Phe_1 (8.98 ppm and 9.04 ppm), thus demonstrating that the *N*-terminus of the peptide is not labeled. Separate resonances for the Lys_{10} α- and ε-amide protons are able to be discerned in the TOCSY spectrum (7.65 ppm and 7.93 ppm), further corroborating labeling of the Lys_{10} ε-amino group.

1D ¹H NMR spectrum (600 MHz, 298 K) of 3.3 mM Lys(BDY FL)$_{10}$-teixobactin in 99.9% DMSO-*d*$_6$.

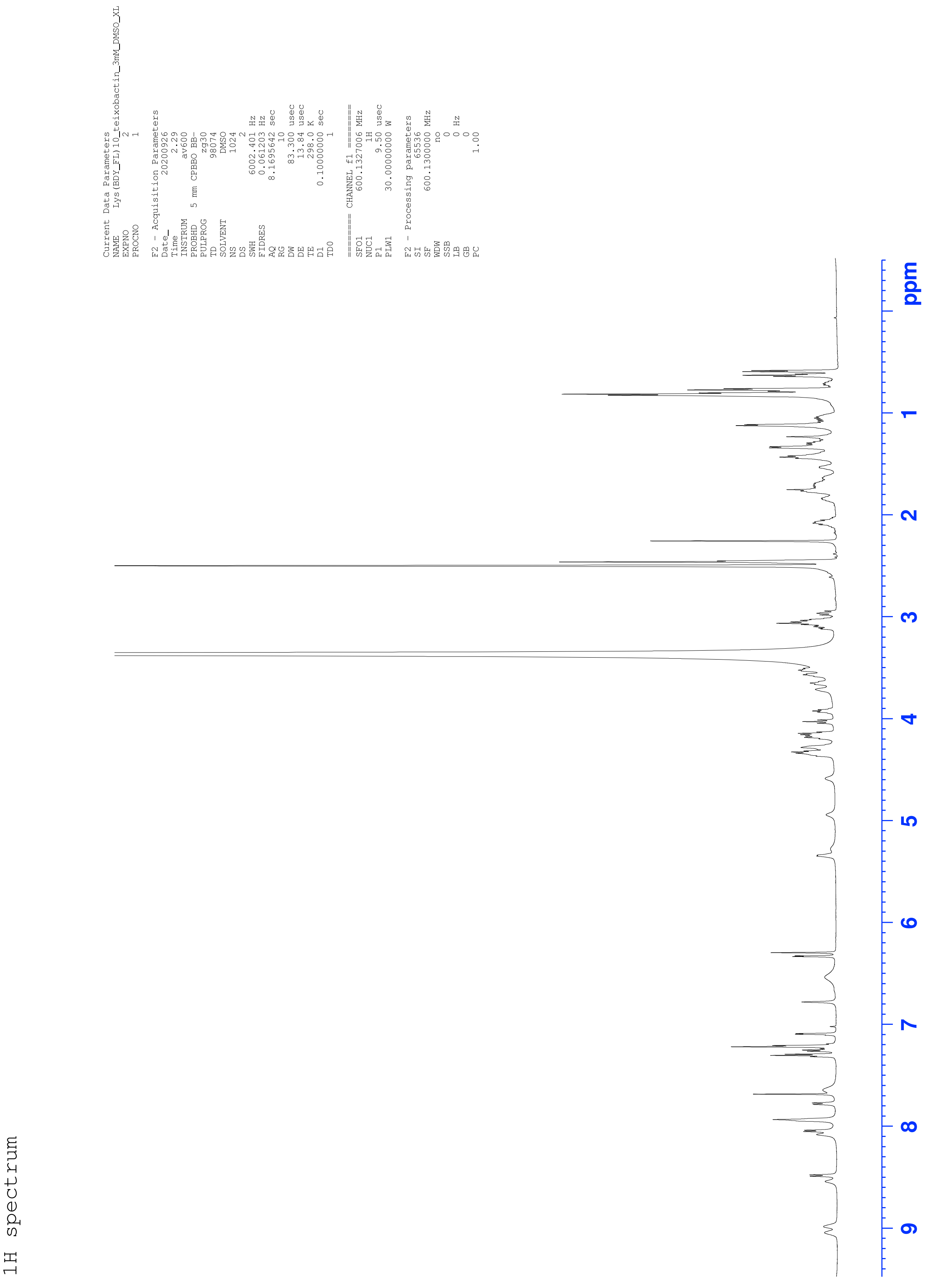

Expansions of ^{1}H NMR spectrum of Lys(BDY FL)$_{10}$-teixobactin, with assignments of key resonances based on TOCSY. These assignments clearly establish that labeling occurs on the side chain of Lys$_{10}$, and not on the α-amino group of N-Me-D-Phe$_1$.

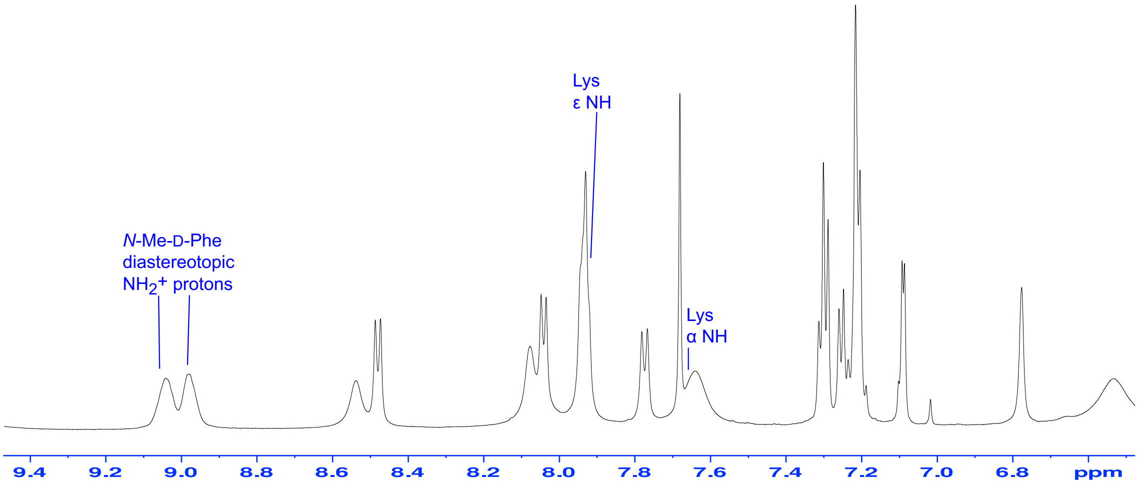

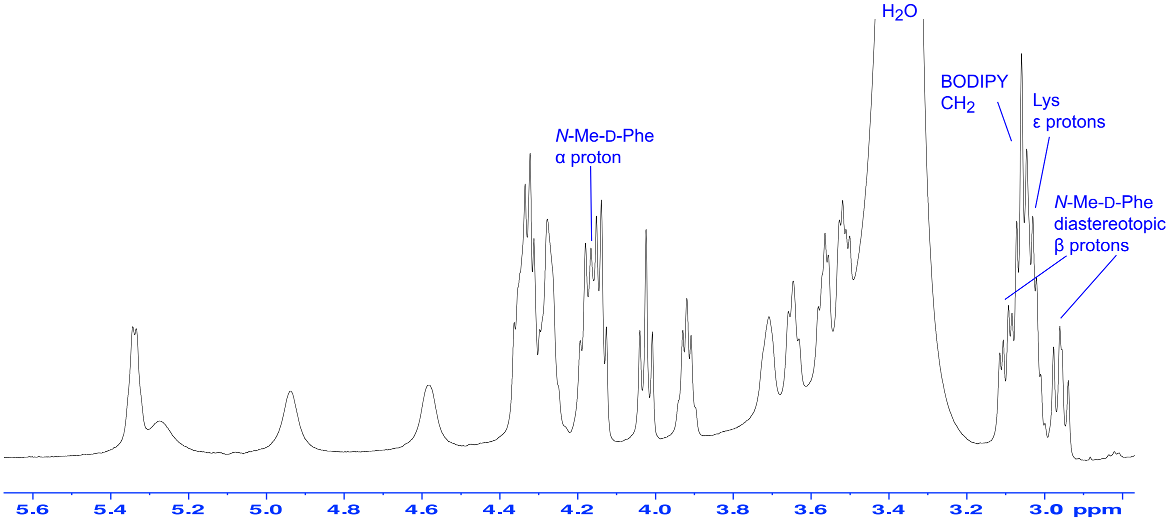

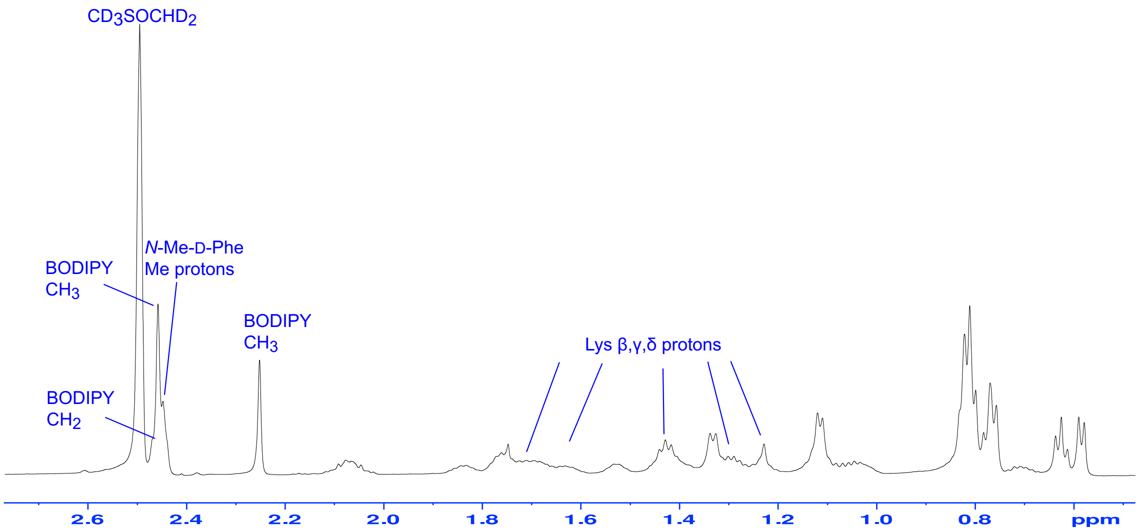

TOCSY spectrum (600 MHz, 150 ms spin-lock mixing time, 298 K) of 3.3 mM Lys(BDY FL)$_{10}$-teixobactin in 99.9% DMSO-d_6 of Lys(BDY FL)$_{10}$-teixobactin, illustrating assignments of key resonances.

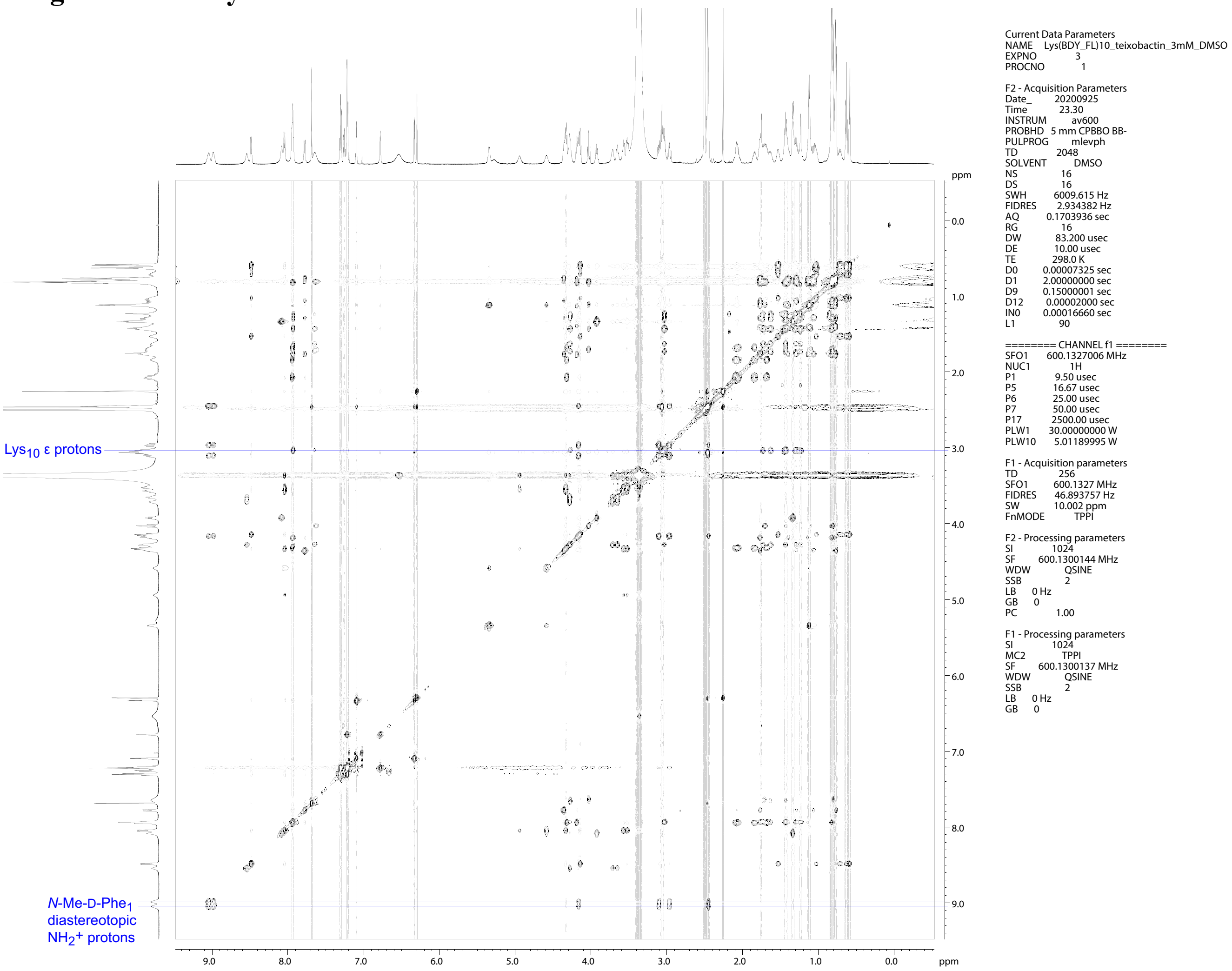

Preparation of DMSO Stock Solutions. A 1 mg/mL DMSO stock solution of each fluorescent teixobactin analogue was prepared gravimetrically by dissolving 1.0 mg of the lyophilized peptide in 1.0 mL of sterile DMSO in an autoclaved Eppendorf tube. The 1 mg/mL DMSO stock solutions were wrapped in black felt and stored in a -20 °C freezer for subsequent experiments.

NOTE: Solutions of fluorescent teixobactin analogues were protected from excessive exposure to light in MIC assays and other experiments by use of an unlit biosafety cabinet, black felt, and minimizing exposure to room lights.

MIC Assays. MIC assays were performed in 96-well plates as previously described, with the exception that optical density measurements were recorded at 750 nm (OD_{750}) to avoid interference by fluorophore absorbance.[3] All MIC assays were performed as three technical replicates and repeated multiple times to ensure reproducibility.

In Vitro PBP2 Transglycosylation Assay using Lipid II. 2 nmol lipid II[4] in in 20 mM MES, 2 mM $MgCl_2$, 2 mM $CaCl_2$ and 0.002% polysorbate 80 (pH 5.5) were incubated with $Lys(BDY FL)_{10}$-teixobactin at molar ratios of 0:1, 1:1, 2:1, 4:1 (antibiotic:lipid II) for 20 min at room temperature. 7.5 µg of purified PBP2-His_6 enzyme[5] were added and the samples were incubated for 2 h at 30 °C, followed by extraction of hydrophobic molecules using an equal volume of *n*-butanol/pyridinium acetate, at pH 4.2 (2:1; *v/v*).[6] Extracts were analyzed by thin-layer chromatography (TLC) using chloroform/methanol/water/ammonia (88:48:10:1, *v/v/v/v*) as the mobile phase[6] and phosphomolybdic acid staining was used to visualize the extracts.[4]

Fluorescence Microscopy Studies. The following were performed as previously described,[3] with the exception that 0.05% polysorbate 80 was not used in steps c and d: (a) Culturing bacteria for imaging; (b) Preparation of 2% agarose beds for imaging bacteria; (c)

Preparation of fluorescent teixobactin solutions for fluorescence microscopy studies; and (d) Staining bacteria for fluorescence microscopy studies. We found that the addition of polysorbate 80 in the probe solutions was not required in the buffer to achieve satisfactory staining of bacteria using the fluorescent teixobactin analogues for the SIM microscopy and FRET microscopy experiments. Sodium phosphate buffer (pH 7.4) was used to prepare all probe solutions and to wash bacteria after staining with the probe solutions.

Structured Illumination Microscopy using the Zeiss Elyra 7. The stained bacteria were immediately imaged at room temperature on a Zeiss Elyra 7 microscope with Lattice SIM[2]. Images were collected with a 63x oil immersion objective lens with numerical aperture of 1.4, using a 50 ms camera exposure (EM CCD camera), with additional digital zoom used as needed. Bacteria (*B. subtilis*) were treated with 1 µg/mL Lys(BDY FL)$_{10}$-teixobactin, Lys(Cy3)$_{10}$-teixobactin, or Lys(Cy5)$_{10}$-teixobactin. Fluorescence micrographs of bacteria treated with Lys(BDY FL)$_{10}$-teixobactin were recorded with excitation at 488 nm and emission between 495–550 nm (using a band-pass filter). Fluorescence micrographs of bacteria treated with Lys(Cy3)$_{10}$-teixobactin were recorded with excitation at 561 nm and emission between 570–620 nm (using a band-pass filter). Fluorescence micrographs of bacteria treated with Lys(Cy5)$_{10}$-teixobactin were recorded with excitation at 642 nm and emission 655 nm and above (using a long-pass filter). The collected SIM images were processed using Zeiss Zen Black software using the SIM processing mode, and the brightness of each micrograph was rescaled to the original micrograph to ensure all pixels were neither over- nor underexposed.

FRET Microscopy. Exponentially growing *B. subtilis* was treated with various mixtures of Lys(Cy3)$_{10}$-teixobactin and Lys(Cy5)$_{10}$-teixobactin (Table S2) or Lys(Cy3)$_{10}$-teixobactin and Lys$_{10}$-teixobactin (Table S3). Using the same staining protocol described above, *B. subtilis*

samples were treated with the probe solutions for 10 min in a shaking incubator at 37 °C. The cells were washed three times using sodium phosphate buffer (without polysorbate 80) and applied to coverslips on agarose beds.

FRET microscopy was performed at room temperature on a 2-channel ISS-ALBA5 scanning confocal microscope equipped with a white laser (NKT SuperK EXTREME), an acousto-optic tunable filter (NKT SELECT) for selecting laser lines. Emission was detected using two avalanche photodiodes (Excelitas Technologies) connected to an ISS FastFLIM unit for single photon counting (and lifetime measurement).

All sets of images were taken using the same parameters: resolution (130 nm per pixel), laser power (~6 μW on the focal plane), pixel dwell time (16 μs) and image dimensions (256 × 256, corresponding approximately to fields of view of 33 × 33 μm). A total of 80 images were taken for the set of FRET experiments, and a total of 45 images were taken for the set of control experiments, with the approximate bacteria count per field of view being between the few dozens to a few hundred.

To ensure selective excitation of the Cy3 probe and not the Cy5, we selected a relatively blue-shifted laser line of 490 nm, thus hitting the lower end of the excitation spectra of the Cy3 (instead of excitation of at λ_{max}, *ca.* 555 nm). Emission of the Cy3 probe was collected using an emission filter centered at 575 nm with a bandwidth of 50 nm. Emission of the Cy5 probe was collected using an emission filter centered at 680 nm with a bandwidth of 40 nm, respectively. When intentionally exciting the Cy5, we selected a laser line at 645 nm.

The intensity-based comparison analysis was performed by simply pooling all the raw pixel intensity values of each experimental condition and plotting the histograms of such

distributions. The total of 125 images yields 8.2 Mpix taking up an area of approximately 0.14 mm^2 and containing in the order of 1×10^4 bacteria in total.

Table S2. Volumes of Lys(Cy3)$_{10}$-teixobactin and Lys(Cy5)$_{10}$-teixobactin stock solutions used to prepare FRET samples.

	100:0 Cy3:Cy5	95:5 Cy3:Cy5	85:5 Cy3:Cy5	75:5 Cy3:Cy5	50:50 Cy3:Cy5	0:100 Cy3:Cy5
**Volume from 1 mg/mL Lys(Cy3)$_{10}$-teixobactin stock**	1 µL	3.8 µL	1.7 µL	1.5 µL	0.5 µL	0 µL
**Volume from 1 mg/mL Lys(Cy5)$_{10}$-teixobactin stock**	0 µL	0.2 µL	0.3 µL	0.5 µL	0.5 µL	1 µL
**Amount of sodium phosphate buffer required to achieve 1 µg/mL**	999 µL	3996 µL	1998 µL	1998 µL	999 µL	999 µL

Table S3. Volumes of Lys(Cy3)$_{10}$-teixobactin and Lys$_{10}$-teixobactin stock solutions used to prepare FRET samples.

	100:0 Cy3: Lys$_{10}$	95:5 Cy3: Lys$_{10}$	85:5 Cy3: Lys$_{10}$	75:5 Cy3: Lys$_{10}$	50:50 Cy3: Lys$_{10}$	0:100 Cy3: Lys$_{10}$
**Volume from 1 mg/mL Lys(Cy3)$_{10}$-teixobactin stock**	1 µL	3.8 µL	1.7 µL	1.5 µL	0.5 µL	0 µL
**Volume from 1 mg/mL Lys$_{10}$-teixobactin stock**	0 µL	0.2 µL	0.3 µL	0.5 µL	0.5 µL	1 µL
**Amount of sodium phosphate buffer required to achieve 1 µg/mL**	999 µL	3996 µL	1998 µL	1998 µL	999 µL	999 µL

Time-Dependent Binding and Bactericidal Activity in B. Subtilis. B. subtilis was grown in Mueller Hinton Broth at 30 °C to the mid-exponential growth phase. Cells were centrifuged for 3 min at 10,000 × *g* and the resulting pellet was resuspended in 10 mM sodium phosphate buffer containing 0.05% (*w/v*) polysorbate 80. The OD_{600} of the cell suspension was adjusted to 0.5 and 3 mL were transferred into a sterile glass tube. Lys$_{10}$-teixobactin and Lys(BDY FL)$_{10}$-teixobactin were added at final concentrations of 3 µg/mL each and the glass vial was placed into an incubator at 30 °C with agitation.

At regular time intervals, 200 µL were taken from the glass vial and centrifuged for 2 min at 10,000 × *g*. The cells were resuspended in 200 µL 0.1 mM propidium iodide in 10 mM sodium phosphate buffer containing 0.05% (*w/v*) polysorbate 80 and then incubated for 6 min at room temperature in the dark. Bacteria were washed twice by centrifugation and subsequent resuspension in 1 mL of 10 mM sodium phosphate buffer containing 0.05% (*w/v*) polysorbate 80 before they were spun down again, resuspended in 50 µL of the same buffer and immediately analyzed by fluorescence microscopy. Therefore, bacteria were spotted onto microscope slides covered with a thin film of 1% (*w/v*) agarose in water. Microscopy was carried out at room temperature using a Zeiss Axio Observer Z1 microscope (Zeiss, Jena, Germany) equipped with HXP 120 V light source and an Axiocam MR3 camera. Standard filter sets were used for BODIPY FL (450–490 nm excitation and 500–550 nm emission) and PI (538–562 nm excitation and 570–640 nm emission) fluorescence.

Image acquisition and analysis were performed with software Zen2 (Zeiss) and Fiji[7] including MicrobeJ v5.13l.[8,9] Exposure settings for all timepoints were optimized using the brightest fluorescence timepoint (120 min) to ensure that histogram settings are identical in all timepoints and micrographs with intense signals are not overexposed.

References for Supporting Information

(1) Guaglianone, G.; Kreutzer, A. G.; Nowick, J. S. Chapter Five - Synthesis and Study of Macrocyclic β-Hairpin Peptides for Investigating Amyloid Oligomers. In *Methods in Enzymology*; Petersson, E. J., Ed.; Academic Press, 2021; Vol. 656, pp 123–168.

(2) Yang, H.; Chen, K. H.; Nowick, J. S. Elucidation of the Teixobactin Pharmacophore. *ACS Chem. Biol.* **2016**, *11* (7), 1823–1826.

(3) Morris, M. A.; Malek, M.; Hashemian, M. H.; Nguyen, B. T.; Manuse, S.; Lewis, K.; Nowick, J. S. A Fluorescent Teixobactin Analogue. *ACS Chem. Biol.* **2020**, *15* (5), 1222–1231.

(4) Schneider, T.; Senn, M. M.; Berger-Bächi, B.; Tossi, A.; Sahl, H.-G.; Wiedemann, I. In Vitro Assembly of a Complete, Pentaglycine Interpeptide Bridge Containing Cell Wall Precursor (Lipid II-Gly5) of Staphylococcus Aureus. *Mol. Microbiol.* **2004**, *53* (2), 675–685.

(5) Schneider, T.; Kruse, T.; Wimmer, R.; Wiedemann, I.; Sass, V.; Pag, U.; Jansen, A.; Nielsen, A. K.; Mygind, P. H.; Raventós, D. S.; Neve, S.; Ravn, B.; Bonvin, A. M. J. J.; De Maria, L.; Andersen, A. S.; Gammelgaard, L. K.; Sahl, H.-G.; Kristensen, H.-H. Plectasin, a Fungal Defensin, Targets the Bacterial Cell Wall Precursor Lipid II. *Science* **2010**, *328* (5982), 1168–1172.
(6) Rick, P. D.; Hubbard, G. L.; Kitaoka, M.; Nagaki, H.; Kinoshita, T.; Dowd, S.; Simplaceanu, V.; Ho, C. Characterization of the Lipid-Carrier Involved in the Synthesis of Enterobacterial Common Antigen (ECA) and Identification of a Novel Phosphoglyceride in a Mutant of Salmonella Typhimurium Defective in ECA Synthesis. *Glycobiology* **1998**, *8* (6), 557–567.
(7) Schindelin, J.; Arganda-Carreras, I.; Frise, E.; Kaynig, V.; Longair, M.; Pietzsch, T.; Preibisch, S.; Rueden, C.; Saalfeld, S.; Schmid, B.; Tinevez, J.-Y.; White, D. J.; Hartenstein, V.; Eliceiri, K.; Tomancak, P.; Cardona, A. Fiji: An Open-Source Platform for Biological-Image Analysis. *Nat. Methods* **2012**, *9* (7), 676–682.
(8) Ducret, A.; Quardokus, E. M.; Brun, Y. V. MicrobeJ, a Tool for High Throughput Bacterial Cell Detection and Quantitative Analysis. *Nature Microbiology* **2016**, *1* (7), 1–7.
(9) https://www.microbej.com/

Supplementary data

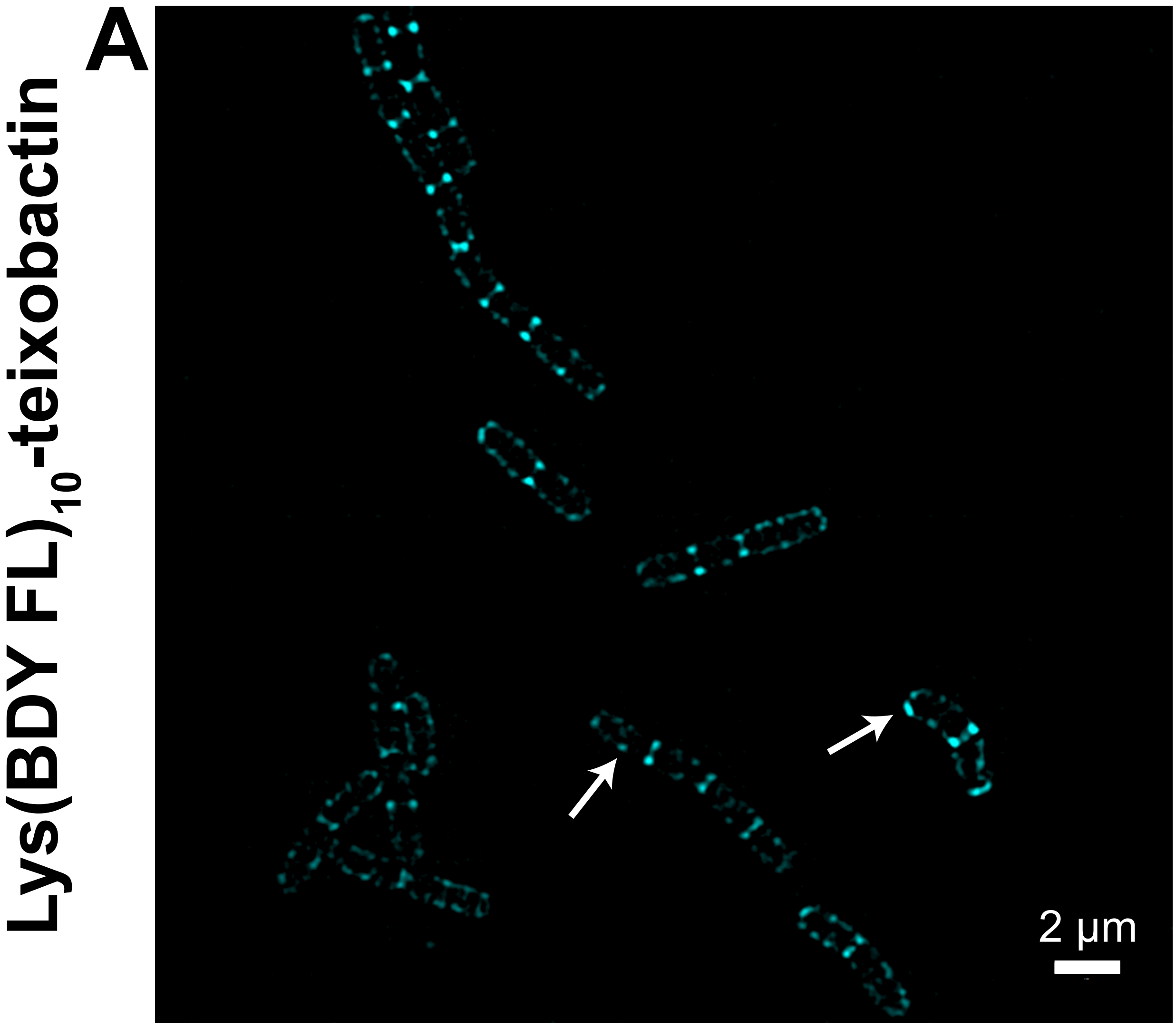

Figure S1A. Expansion of Lys(BDY FL)$_{10}$-teixobactin micrograph from Figure 3A.

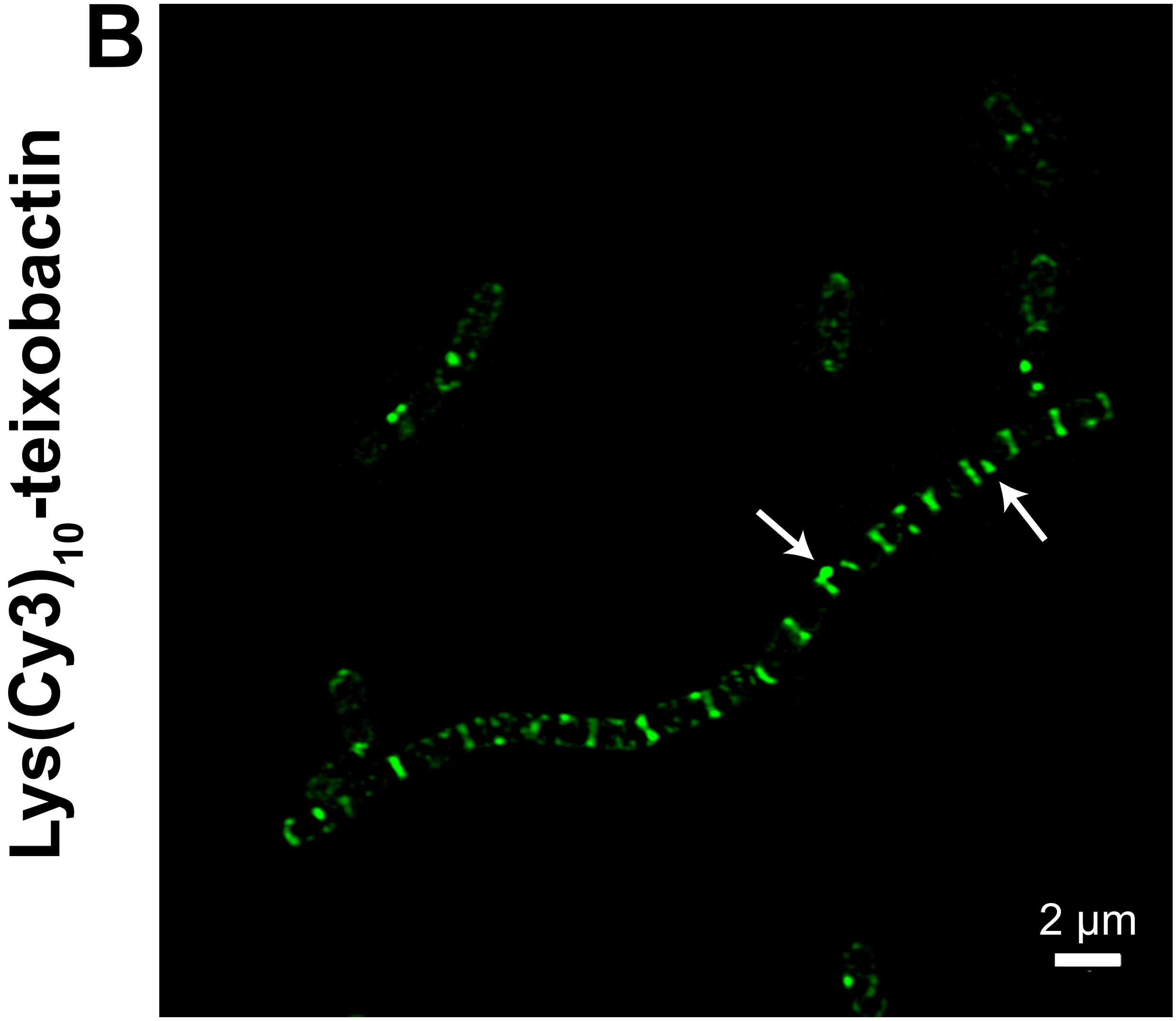

Figure S1B. Expansion of Lys(Cy3)$_{10}$-teixobactin micrograph from Figure 3B.

C

$Lys(Cy5)_{10}$-teixobactin

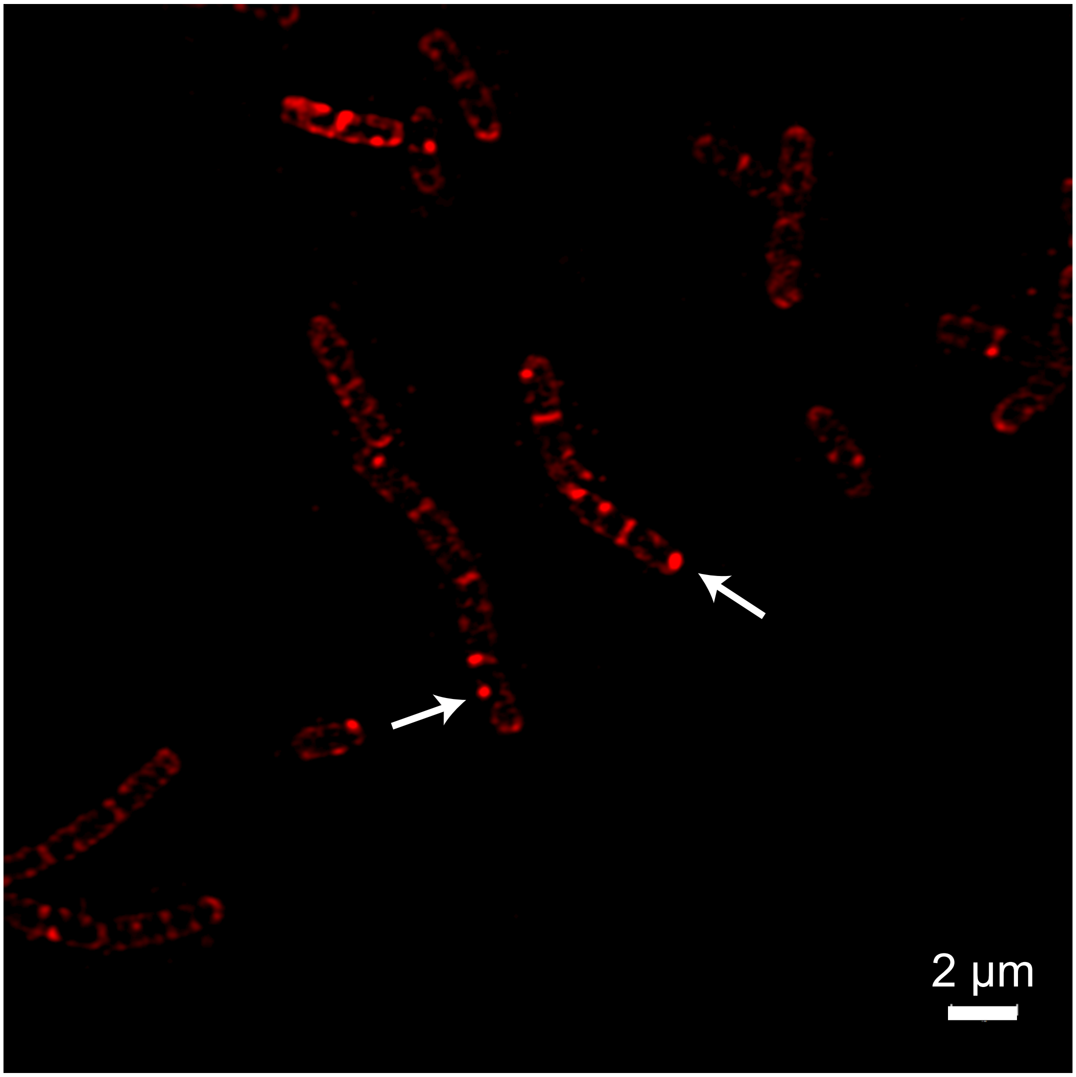

Figure S1C. Expansion of $Lys(Cy5)_{10}$-teixobactin micrograph from Figure 3C.

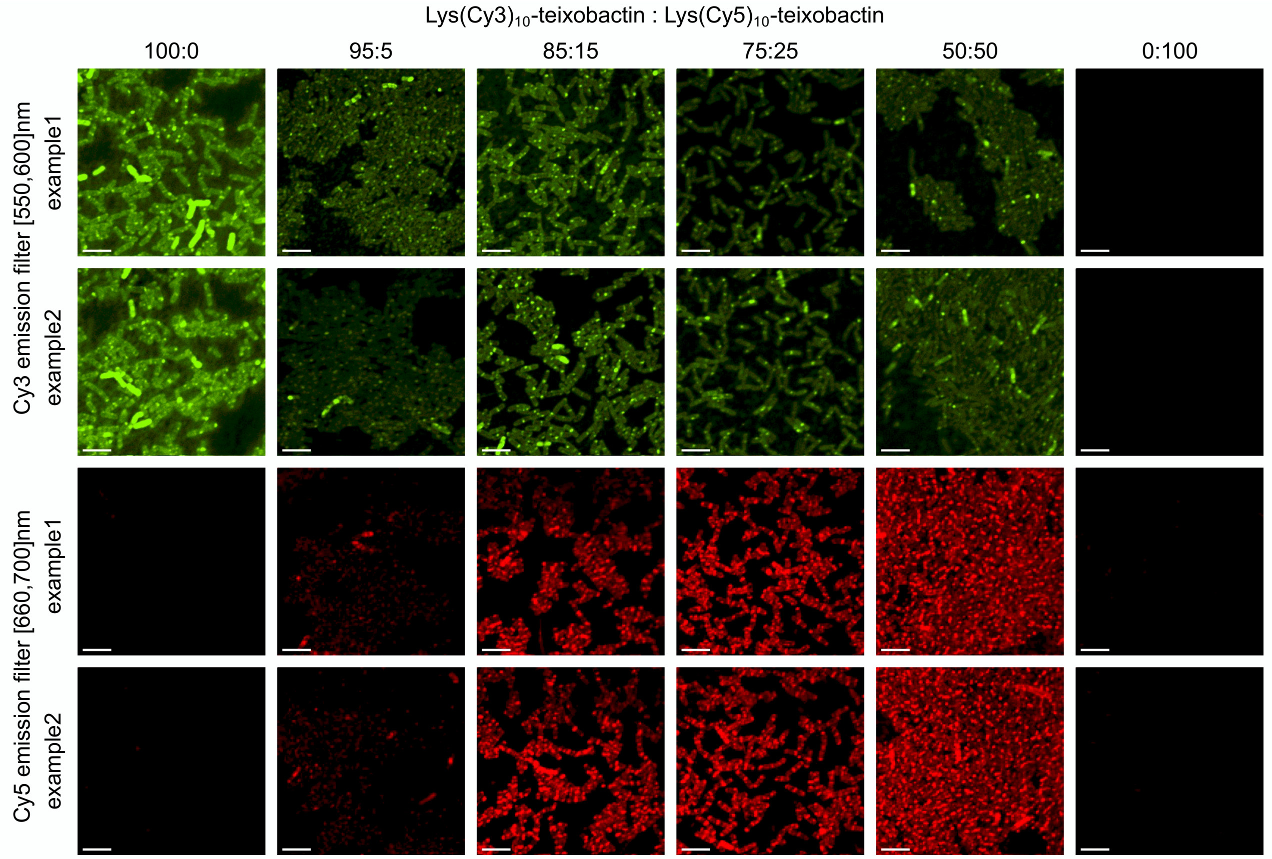

Figure S2. Additional representative micrographs from the Lys(Cy3)$_{10}$-teixobactin/Lys(Cy5)$_{10}$-teixobactin FRET experiments for each relative concentration (scale bars are 5 μm). Micrographs were acquired and processed using the same settings, to allow comparisons in intensity at varying ratios of Lys(Cy3)$_{10}$-teixobactin and Lys(Cy5)$_{10}$-teixobactin.

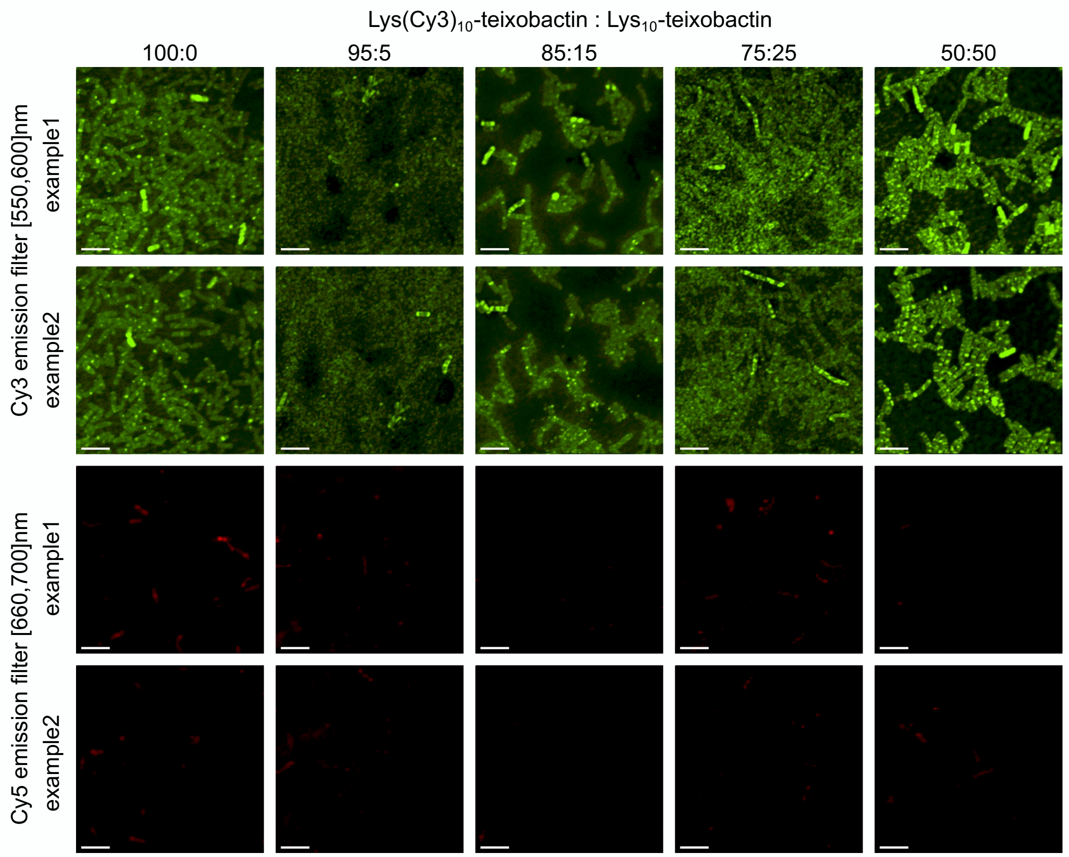

Figure S3. Additional representative micrographs from the Lys(Cy3)$_{10}$-teixobactin/Lys$_{10}$-teixobactin FRET control experiments for each relative concentration (scale bars are 5 µm). Micrographs were acquired and processed using the same settings, to allow comparisons in intensity at varying ratios of Lys(Cy3)$_{10}$-teixobactin and Lys$_{10}$-teixobactin.

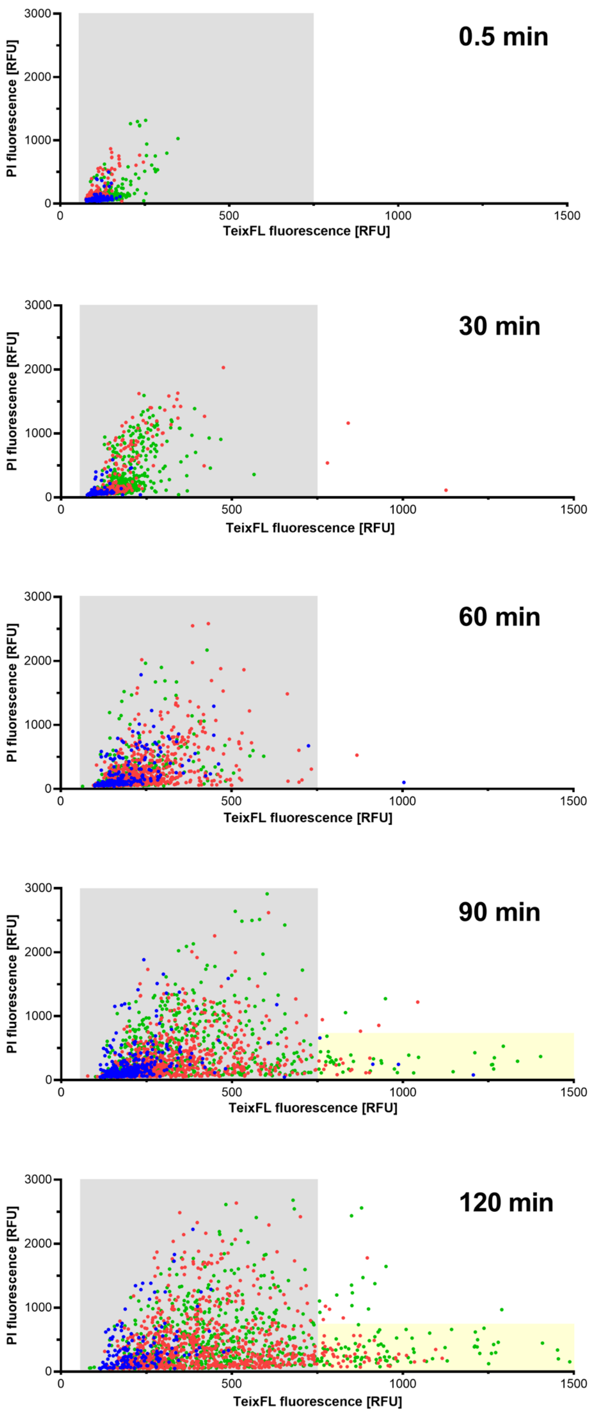

Figure S4. Analysis of the PI fluorescence distribution as a function of Lys(BDY FL)$_{10}$-teixobactin fluorescence in individual *B. subtilis* cells at different time points. Colored dots represent the bacteria measured in three biological independent experiments. The gray boxes highlight bacteria with fluorescence intensities < 3000 RFU PI and ≤ 750 RFU BODIPY FL. The yellow boxes highlight cells with fluorescence intensities > 750 RFU PI and ≤1500 RFU BODIPY FL.

Characterization data

Lys$_{10}$-teixobactin

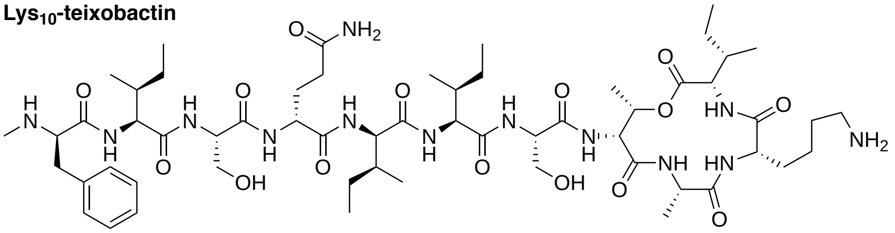

Chemical Formula: $C_{58}H_{97}N_{13}O_{15}$
Exact Mass: 1215.72

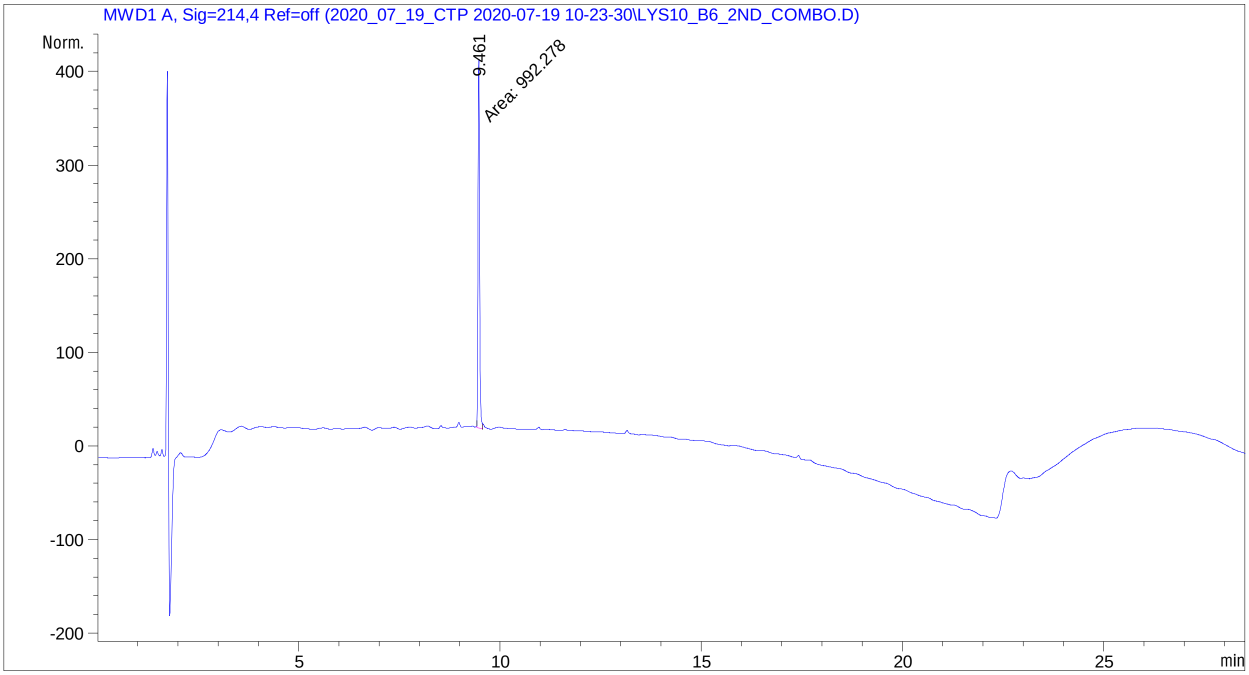


```
Signal 1: MWD1 A, Sig=214,4 Ref=off

Peak RetTime Type  Width     Area      Height     Area
  #   [min]        [min]   [mAU*s]     [mAU]        %
----|-------|----|-------|----------|----------|--------|
   1   9.461 MM    0.0462  992.27832  358.02289 100.0000

Totals :                   992.27832  358.02289
```

Calculated mass for Lys$_{10}$-teixobactin: $[M+H]^+$ = 1216.73

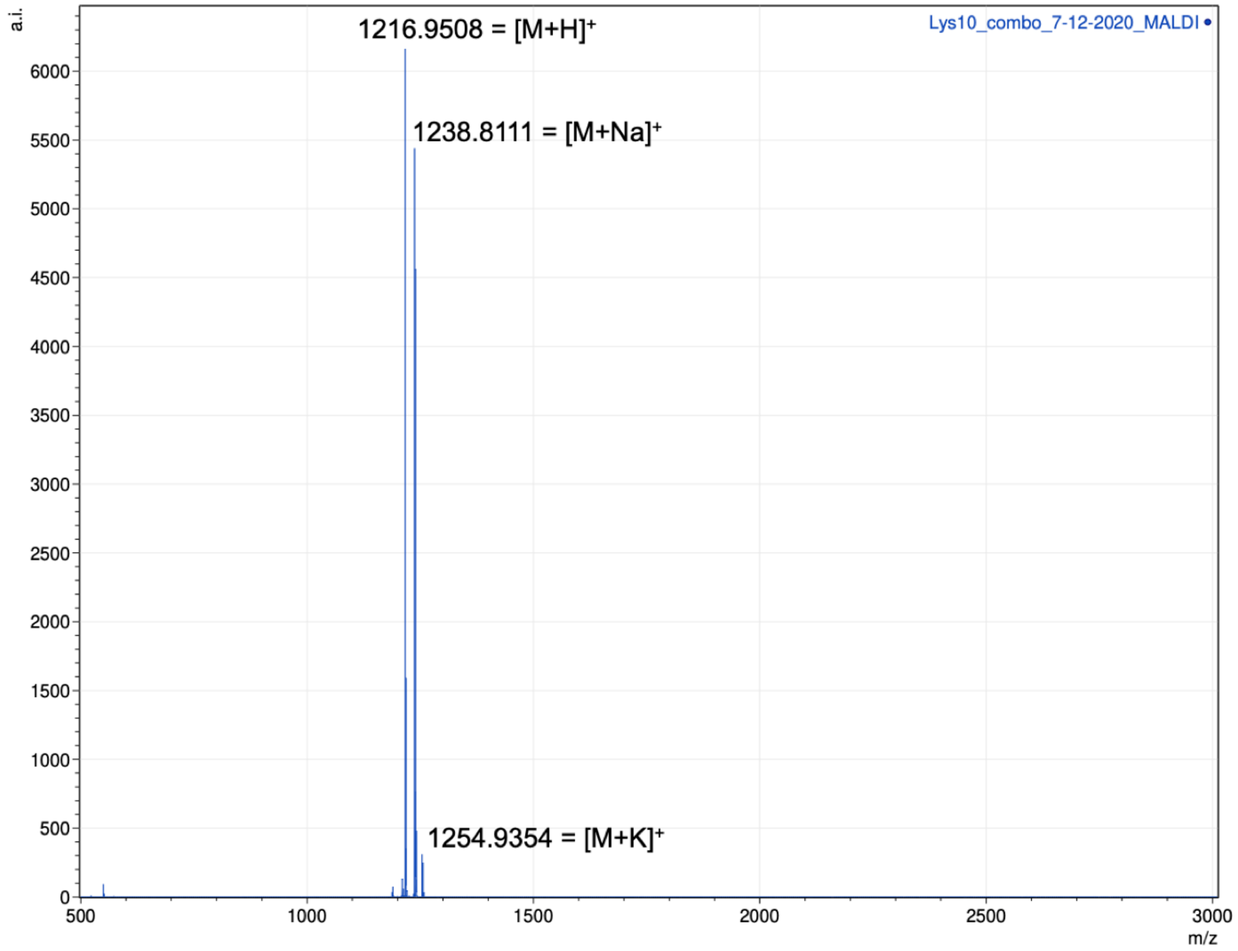

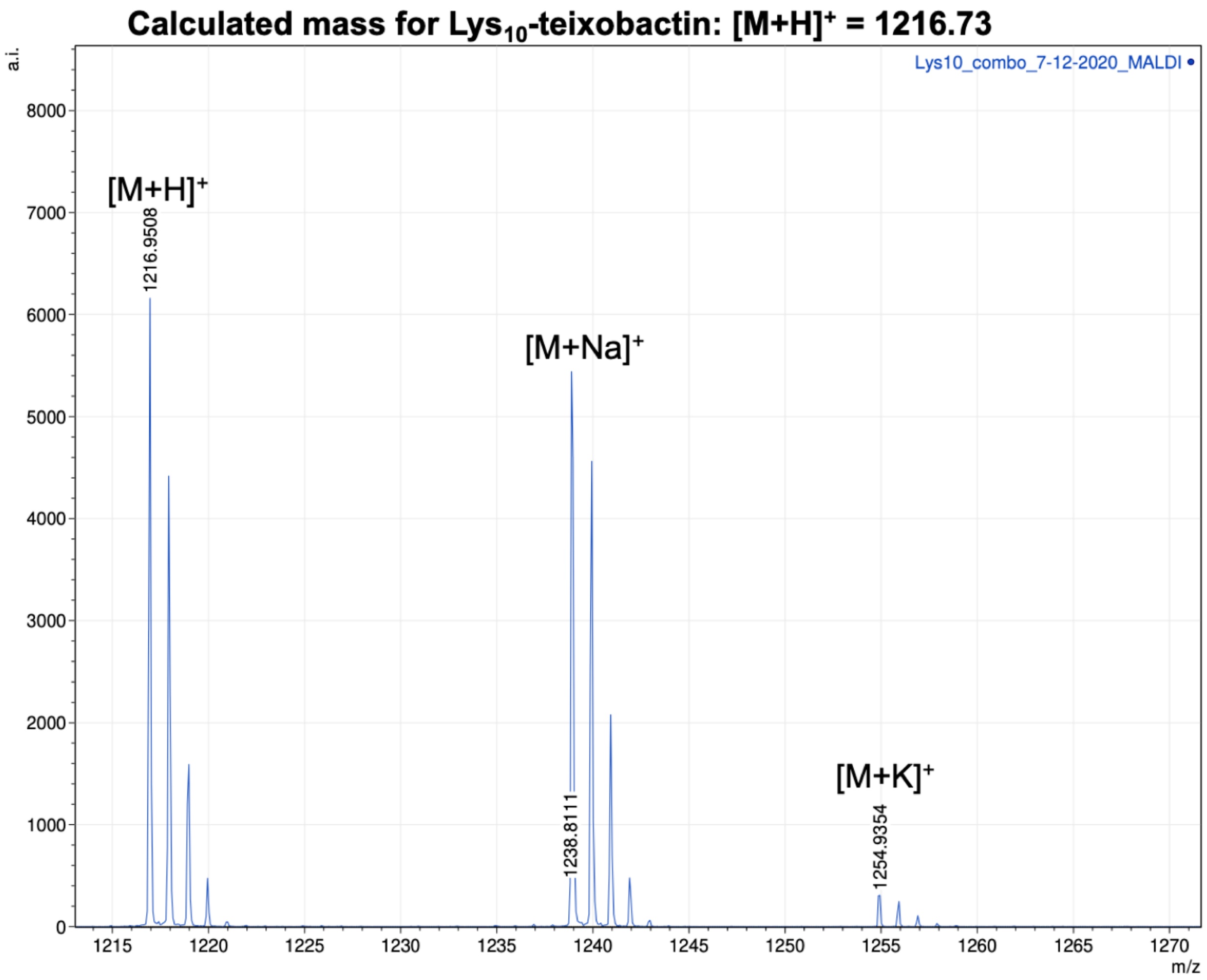
Calculated mass for Lys10-teixobactin: [M+H]+ = 1216.73
Lys10_combo_7-12-2020_MALDI
a.i.
[M+H]+
1216.9508
[M+Na]+
1238.8111
[M+K]+
1254.9354
m/z

Lys(BDY FL)$_{10}$-teixobactin

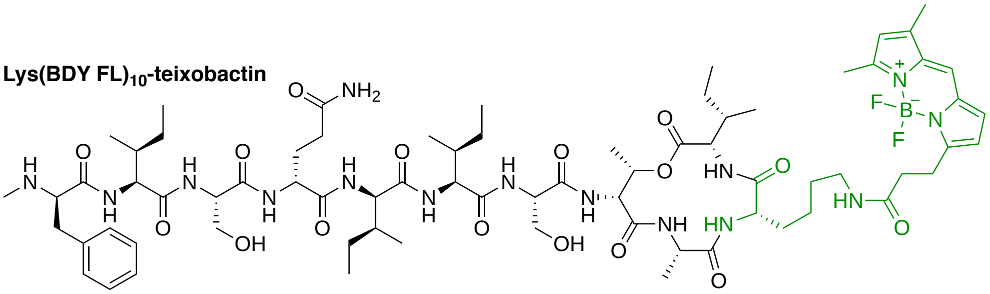

Chemical Formula: $C_{72}H_{110}BF_2N_{15}O_{16}$
Exact Mass: 1489.83

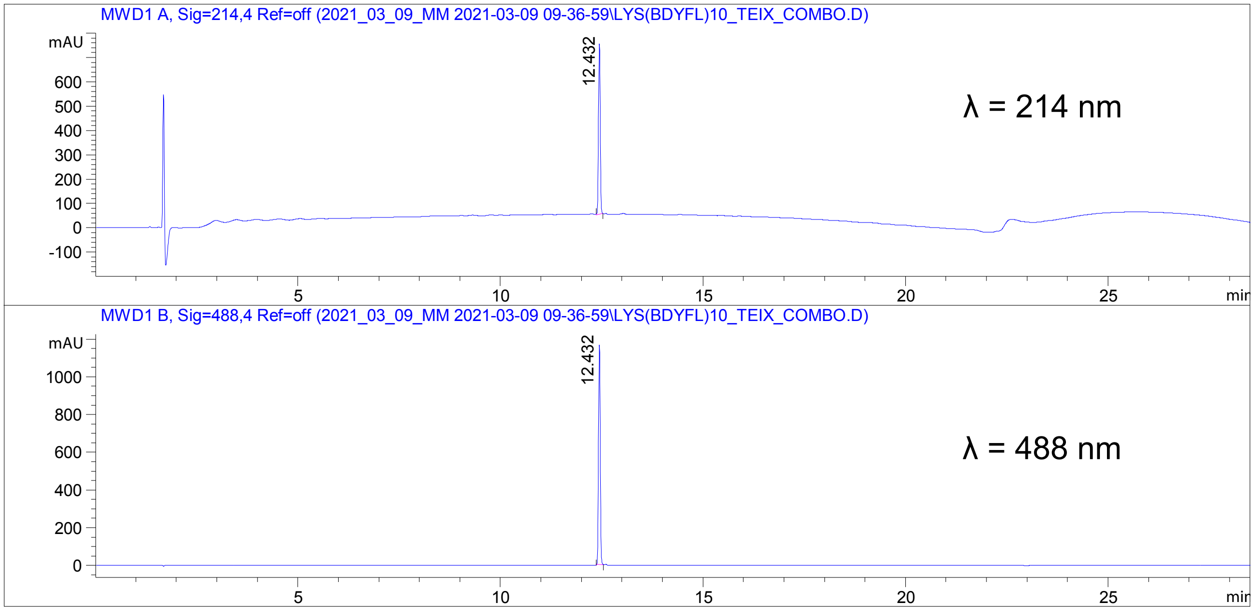

Signal 1: MWD1 A, Sig=214,4 Ref=off

Peak #	RetTime [min]	Type	Width [min]	Area [mAU*s]	Height [mAU]	Area %
1	12.432	MM	0.0484	2043.48901	703.70245	100.0000

ESI-MS of Lys(BDY FL)$_{10}$-teixobactin (LRMS)

Calculated mass of Lys(BDY FL)$_{10}$-teixobactin: $[M+H]^+$ = 1490.83

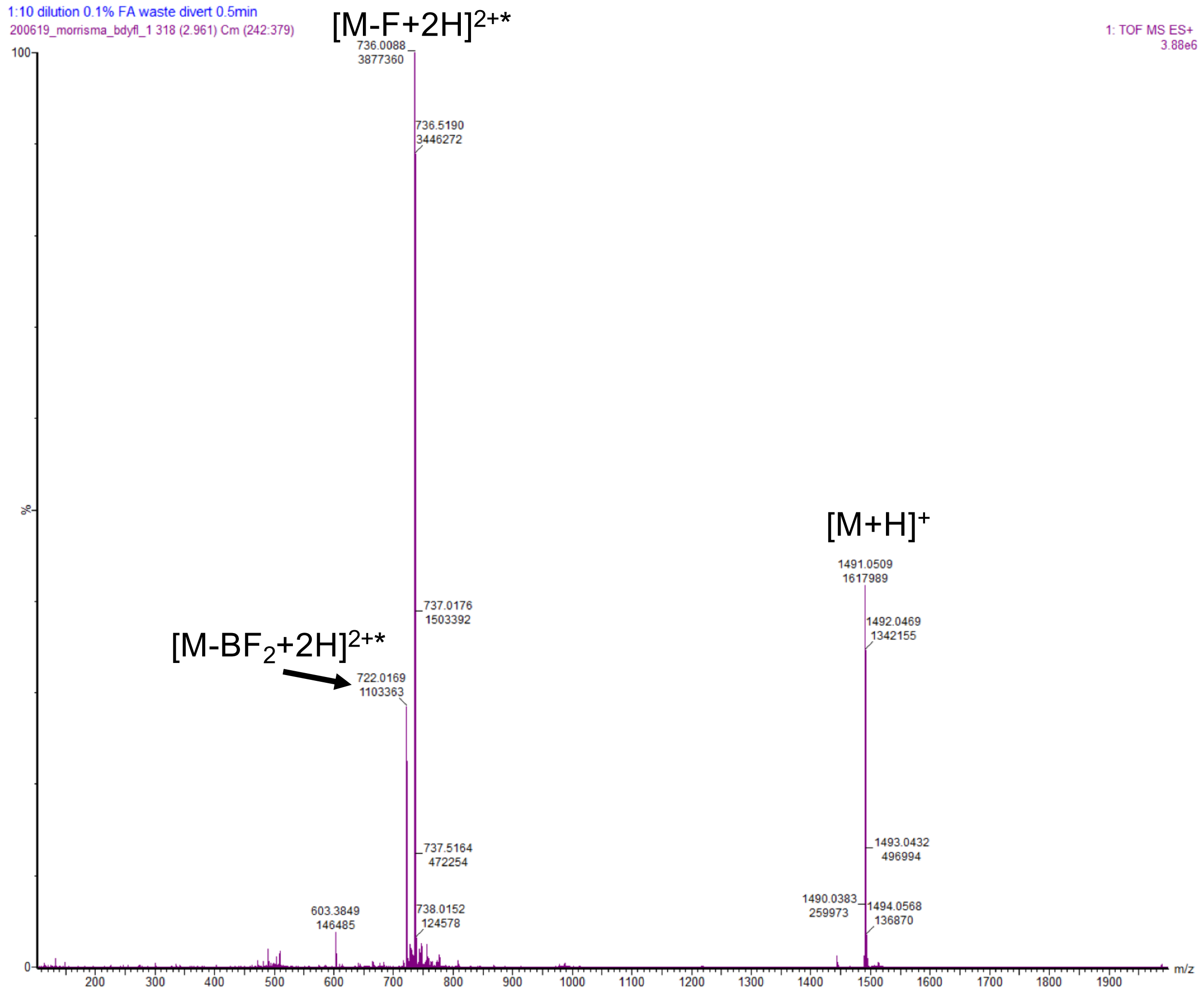

Note: electrospray ionization of BODIPY FL can result in the fragmentation of BODIPY FL, generating the loss of fluoride (F^-) and $(BF_2)^+$ ions, which is why $[M\text{-}F+2H]^{2+}$ = 736 and $[M\text{-}BF_2+2H]^{2+}$ = 722 are observed.

HRMS (ESI-TOF) of Lys(BDY FL)$_{10}$-teixobactin

HRMS (ESI-TOF) *m/z*: [M-F+H]$^{2+}$ calcd for $C_{72}H_{111}BFN_{15}O_{16}$ 735.9200 found 735.9200

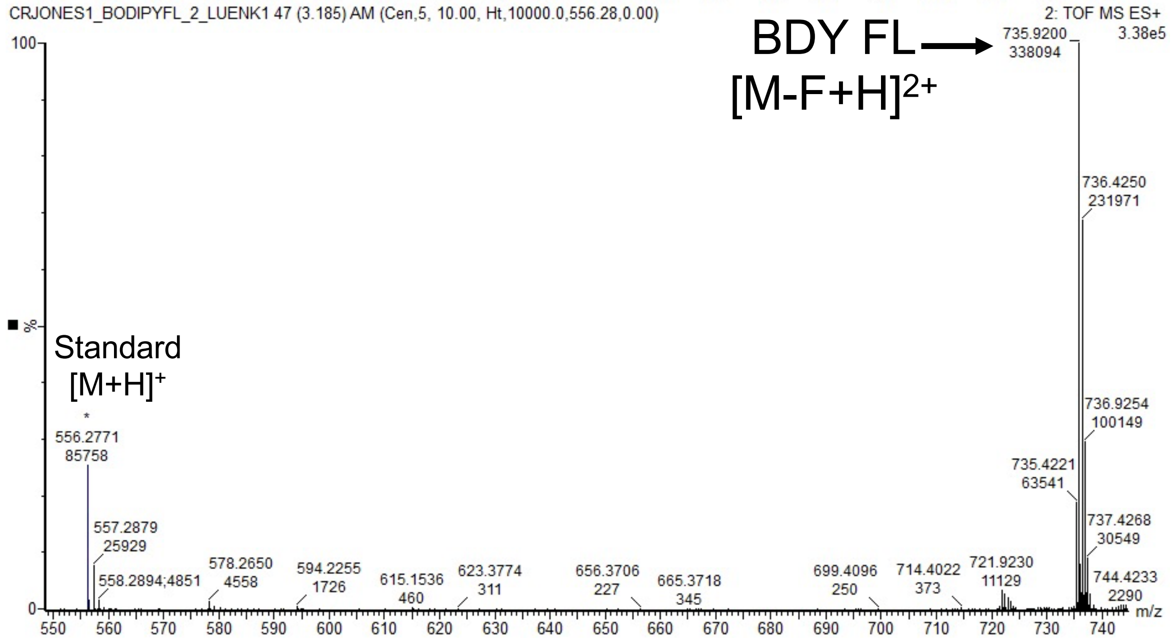

HRMS on Lys(BDY FL)$_{10}$-teixobactin was performed on a Waters Synapt G2 using Leucine Enkephalin as the calibrant.

Note: electrospray ionization of BODIPY FL can result in the fragmentation of BODIPY FL, generating the loss of fluoride (F^-), which is why [M-F+H]$^{2+}$ = 735.9200 is observed.

HRMS (ESI-TOF) of Lys(BDY FL)$_{10}$-teixobactin

HRMS (ESI-TOF) *m/z*: $[M\text{-}F\text{+}H]^{2+}$ calcd for $C_{72}H_{111}BFN_{15}O_{16}$ 735.9200 found 735.9200

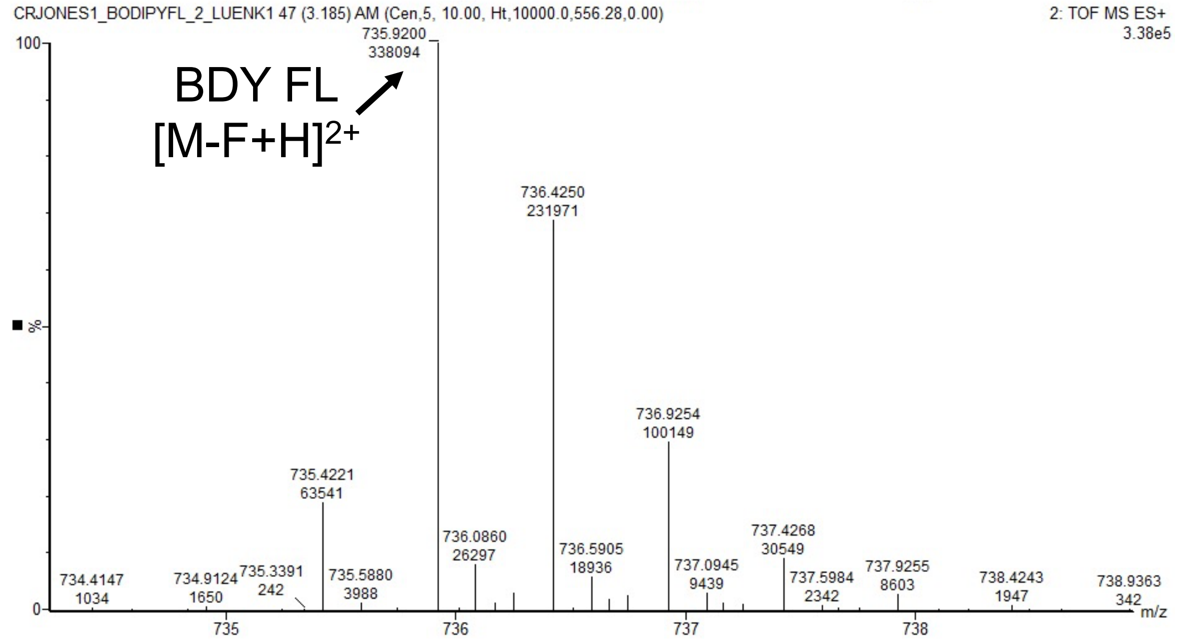

HRMS on Lys(BDY FL)$_{10}$-teixobactin was performed on a Waters Synapt G2 using Leucine Enkephalin as the calibrant.

Note: electrospray ionization of BODIPY FL can result in the fragmentation of BODIPY FL, generating the loss of fluoride (F^-), which is why $[M\text{-}F\text{+}H]^{2+}$ = 735.9200 is observed.

Lys(Cy3)$_{10}$-teixobactin

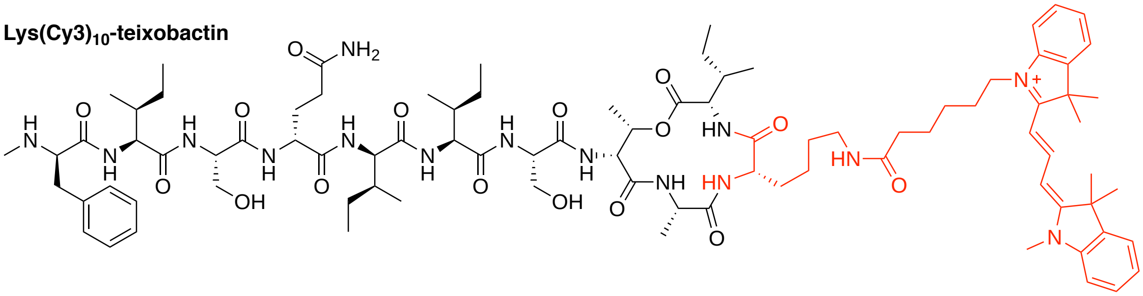

Chemical Formula: $C_{88}H_{132}N_{15}O_{16}^{+}$
Exact Mass: 1655.00

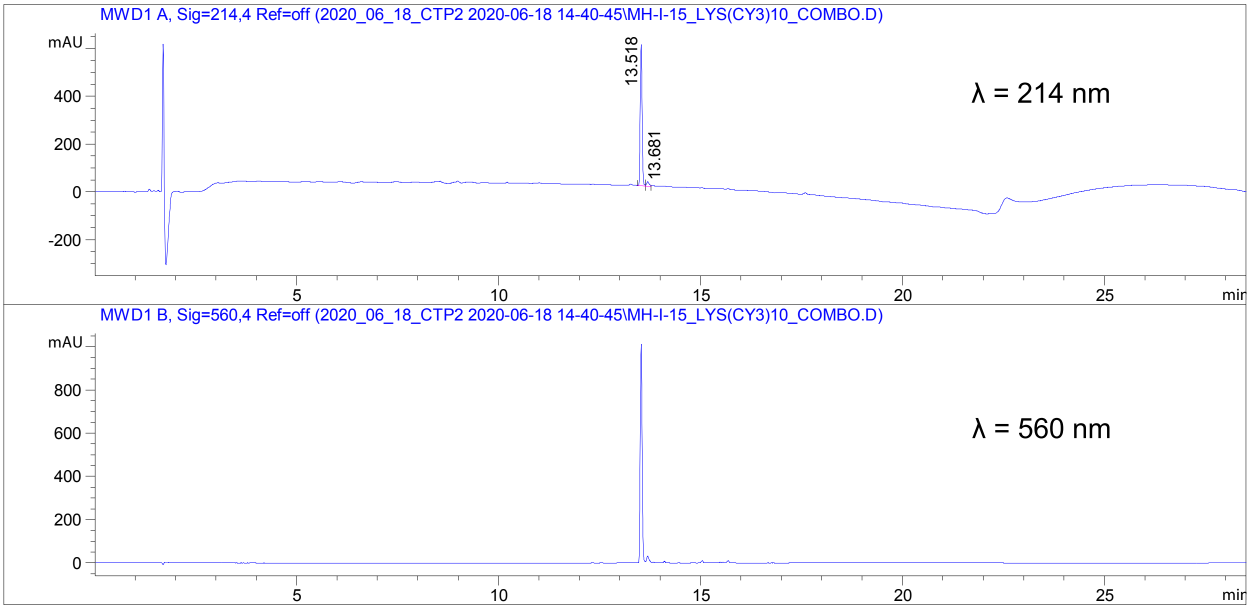

Peak #	RetTime [min]	Type	Width [min]	Area [mAU*s]	Height [mAU]	Area %
1	13.518	MF	0.0520	1852.17371	593.26880	95.5362
2	13.681	FM	0.0761	86.54102	18.95244	4.4638

MALDI-TOF mass spectrum of Lys(Cy3)$_{10}$-teixobactin (LRMS)

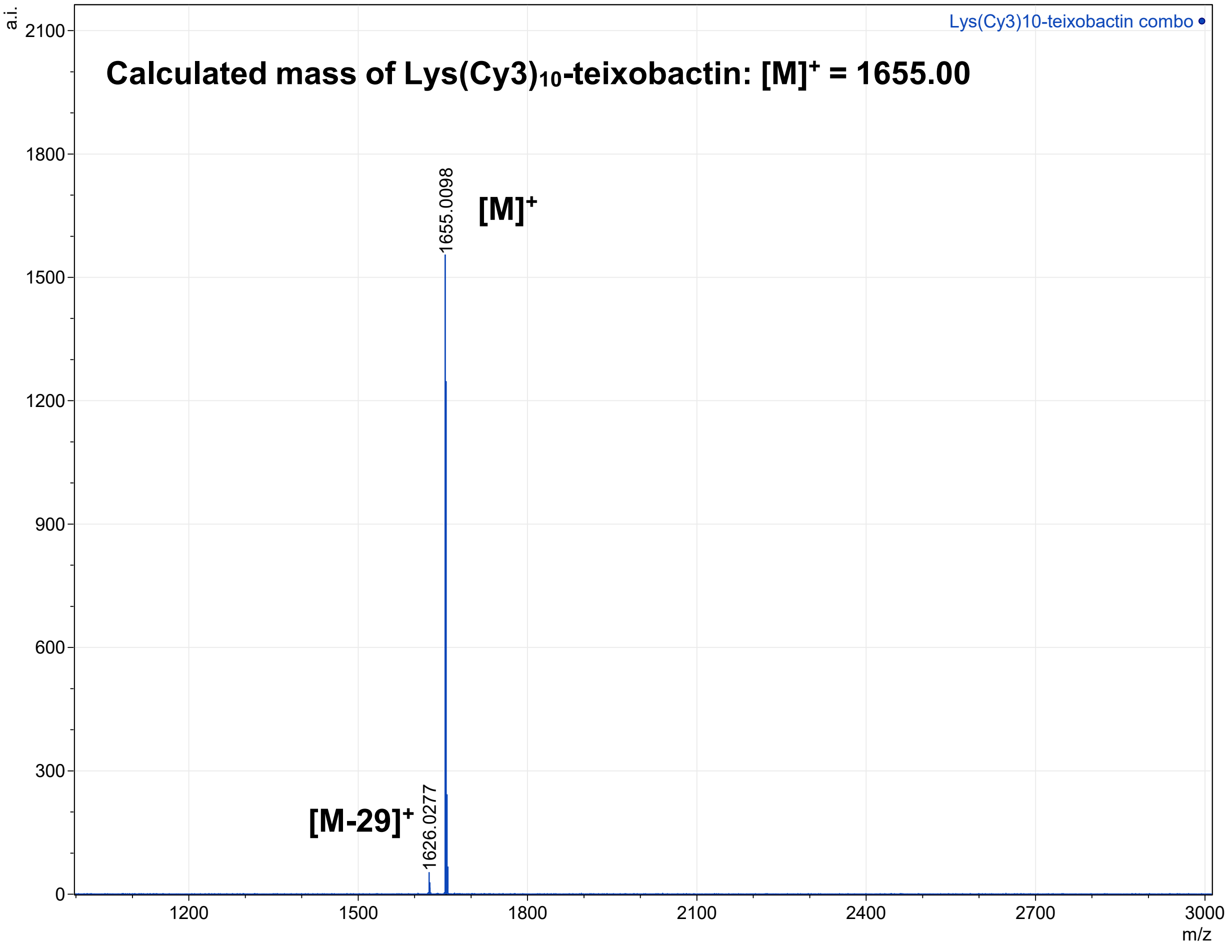

MALDI-TOF mass spectrum of Lys(Cy3)$_{10}$-teixobactin (LRMS)

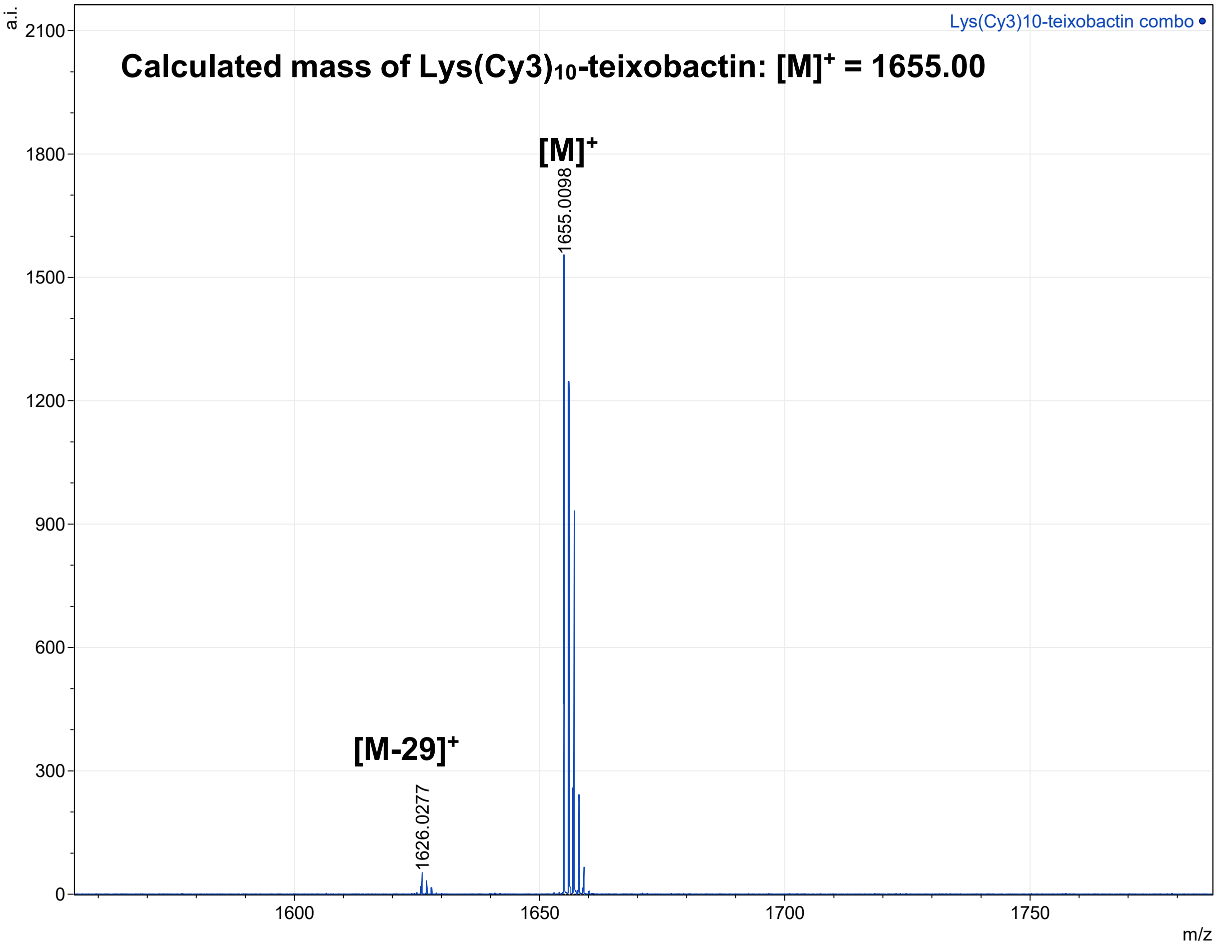

HRMS (ESI-TOF) of Lys(Cy3)$_{10}$-teixobactin

HRMS (ESI-TOF) *m/z*: $[M+H]^{2+}$ calcd for $C_{88}H_{133}N_{15}O_{16}$ 828.0026 found 827.9991

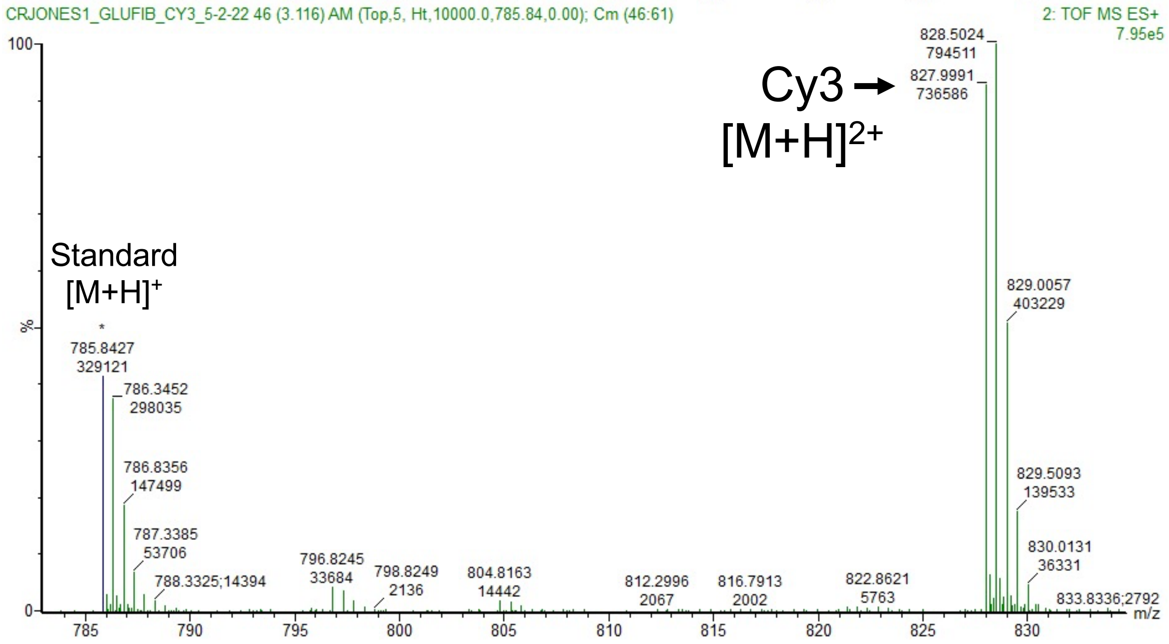

HRMS on Lys(Cy3)$_{10}$-teixobactin was performed on a Waters Synapt G2 using [Glu1]-Fibrinopeptide B as the calibrant.

HRMS (ESI-TOF) of Lys(Cy3)$_{10}$-teixobactin

HRMS (ESI-TOF) *m/z*: $[M+H]^{2+}$ calcd for $C_{88}H_{133}N_{15}O_{16}$ 828.0026 found 827.9991

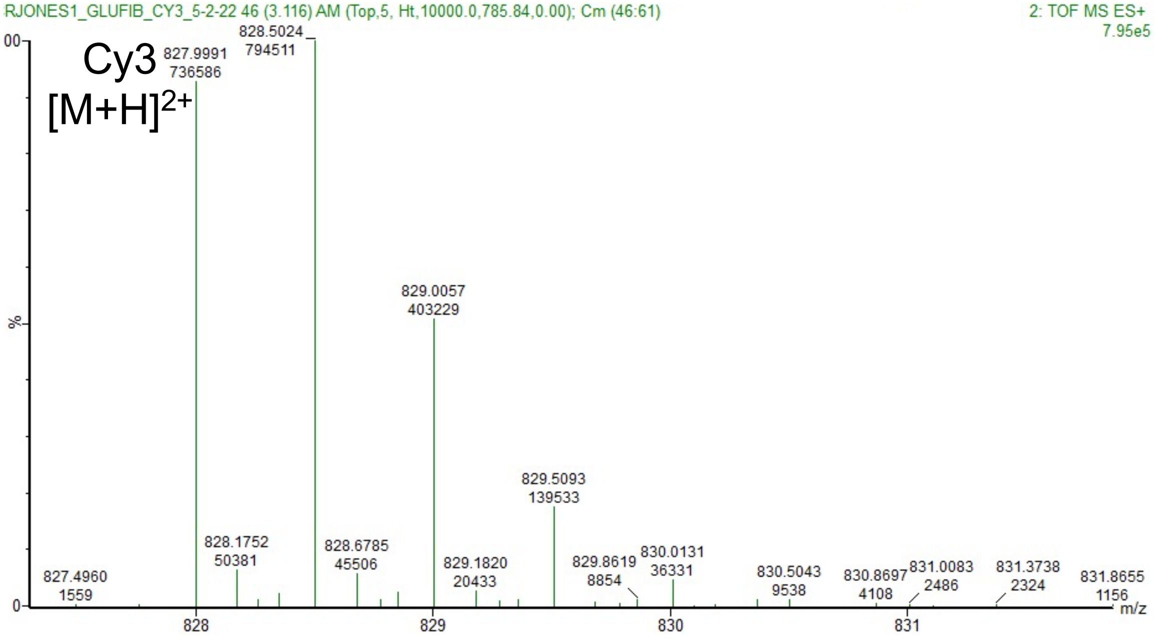

HRMS on Lys(Cy3)$_{10}$-teixobactin was performed on a Waters Synapt G2 using [Glu1]-Fibrinopeptide B as the calibrant.

Lys(Cy5)$_{10}$-teixobactin

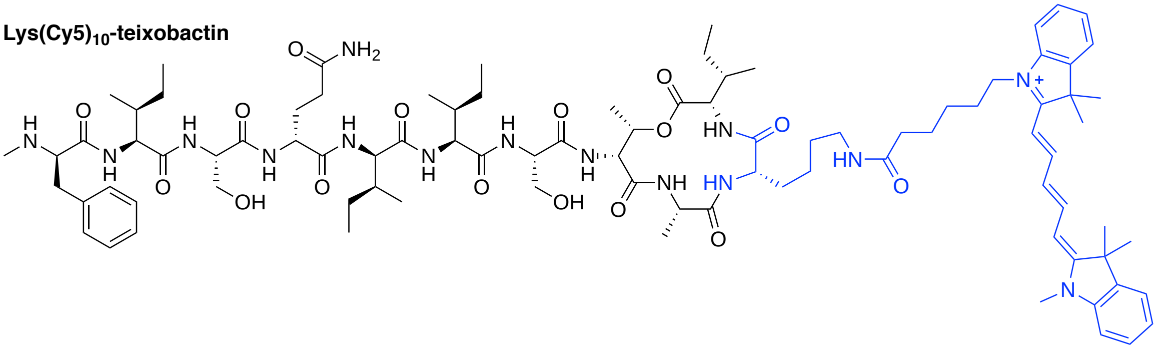

Chemical Formula: $C_{90}H_{134}N_{15}O_{16}^{+}$
Exact Mass: 1681.01

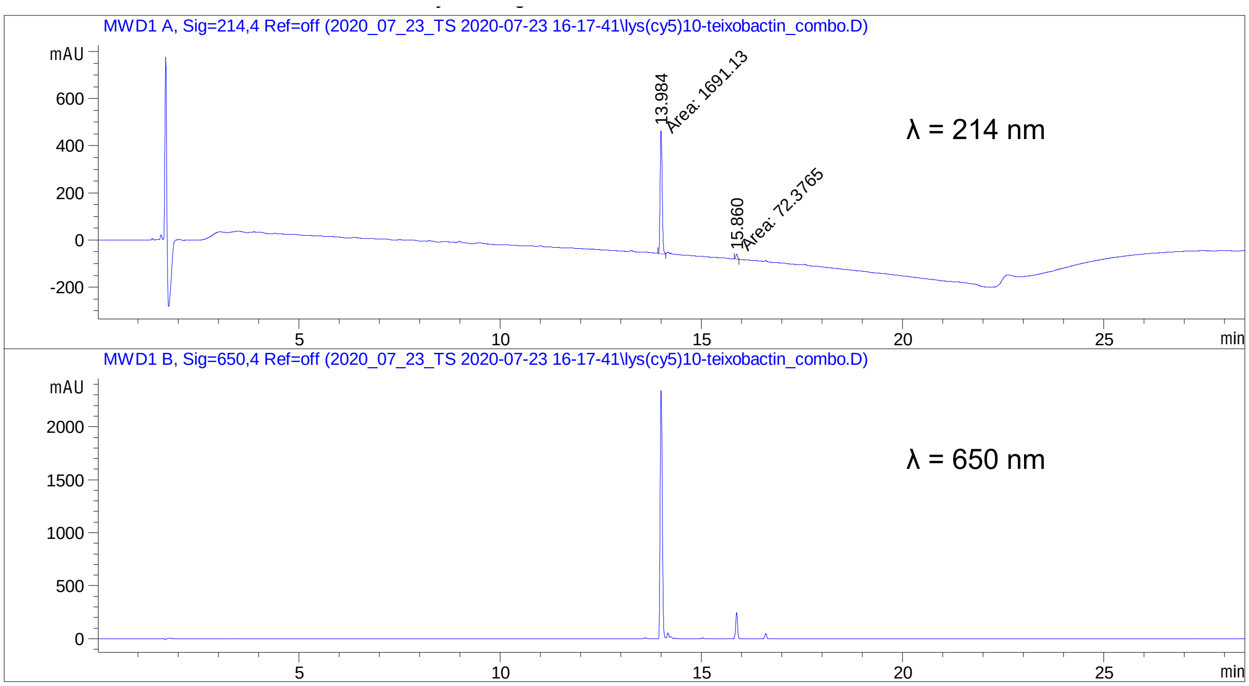

Signal 1: MWD1 A, Sig=214,4 Ref=off

Peak #	RetTime [min]	Type	Width [min]	Area [mAU*s]	Height [mAU]	Area %
1	13.984	MM	0.0536	1691.12512	526.08240	95.8959
2	15.860	MM	0.0540	72.37655	22.33026	4.1041

Totals : 1763.50167 548.41266

MALDI-TOF mass spectrum of Lys(Cy5)$_{10}$-teixobactin (LRMS)

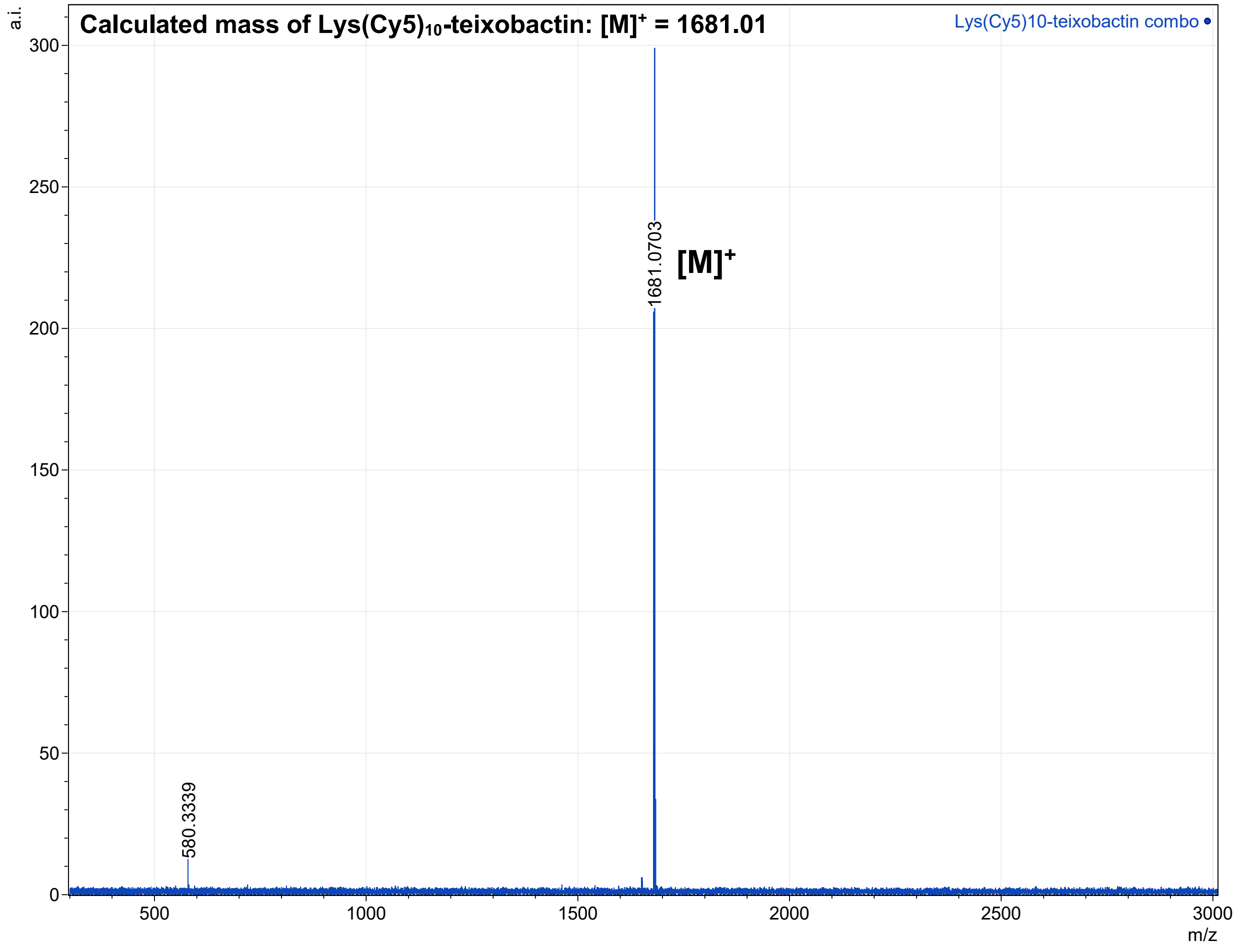

MALDI-TOF mass spectrum of Lys(Cy5)$_{10}$-teixobactin (LRMS)

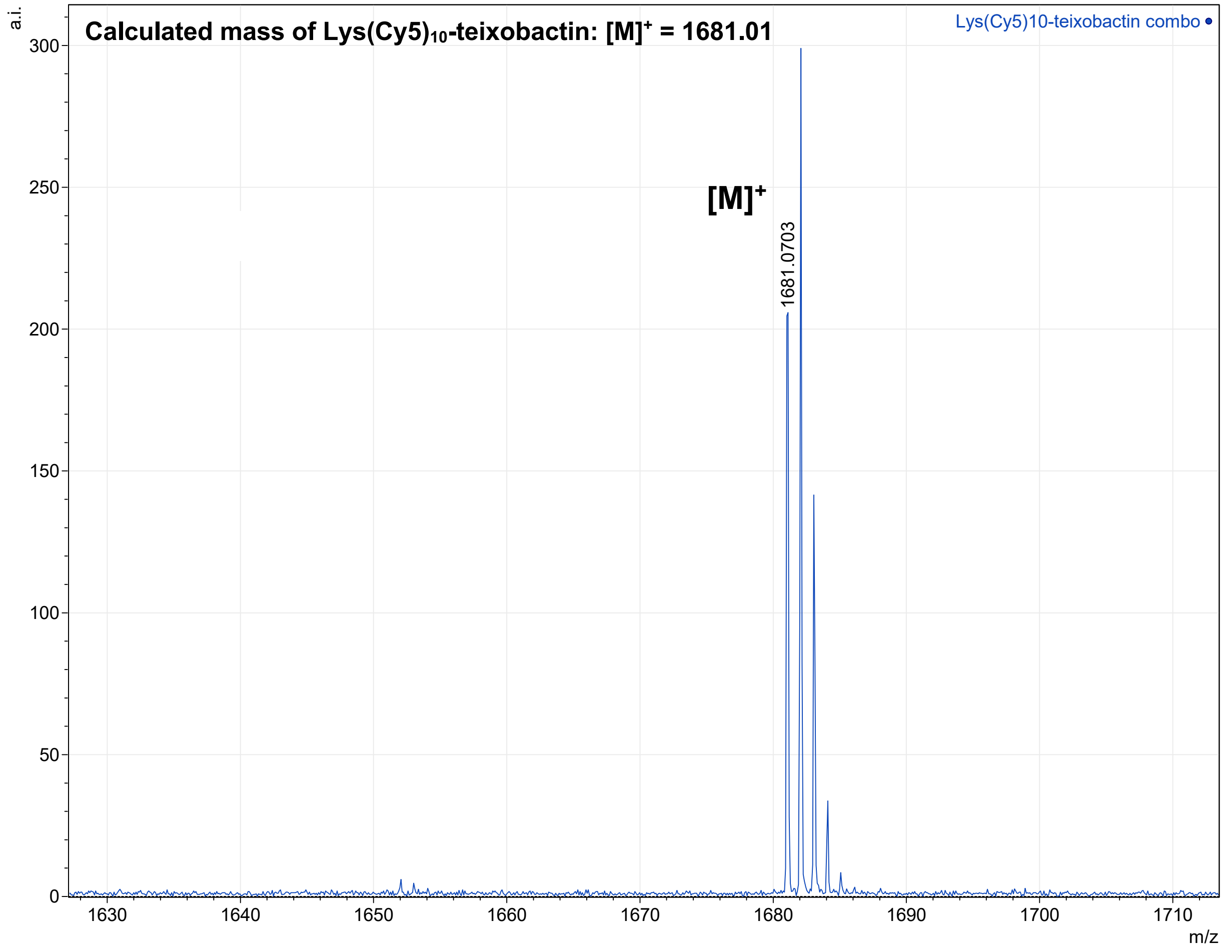

HRMS (ESI-TOF) of Lys(Cy5)$_{10}$-teixobactin

HRMS (ESI-TOF) *m/z*: $[M+H]^{2+}$ calcd for $C_{90}H_{135}N_{15}O_{16}$ 841.0100 found 841.0066

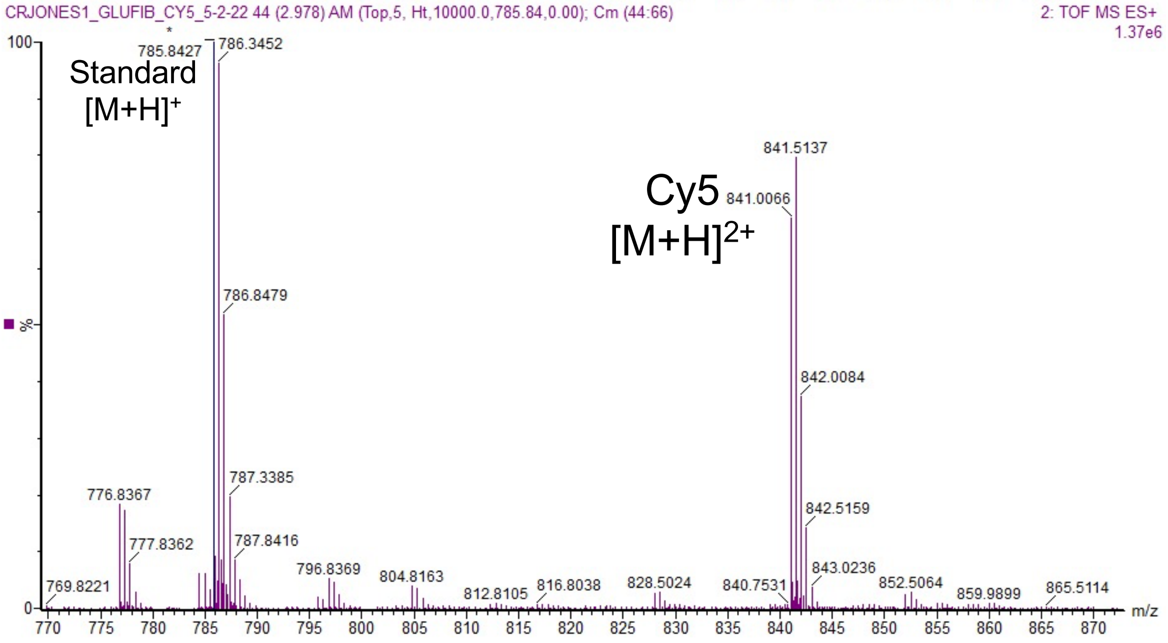

HRMS on Lys(Cy5)$_{10}$-teixobactin was performed on a Waters Synapt G2 using [Glu1]-Fibrinopeptide B as the calibrant.

HRMS (ESI-TOF) of Lys(Cy5)$_{10}$-teixobactin

HRMS (ESI-TOF) *m/z*: $[M+H]^{2+}$ calcd for $C_{90}H_{135}N_{15}O_{16}$ 841.0100 found 841.0066

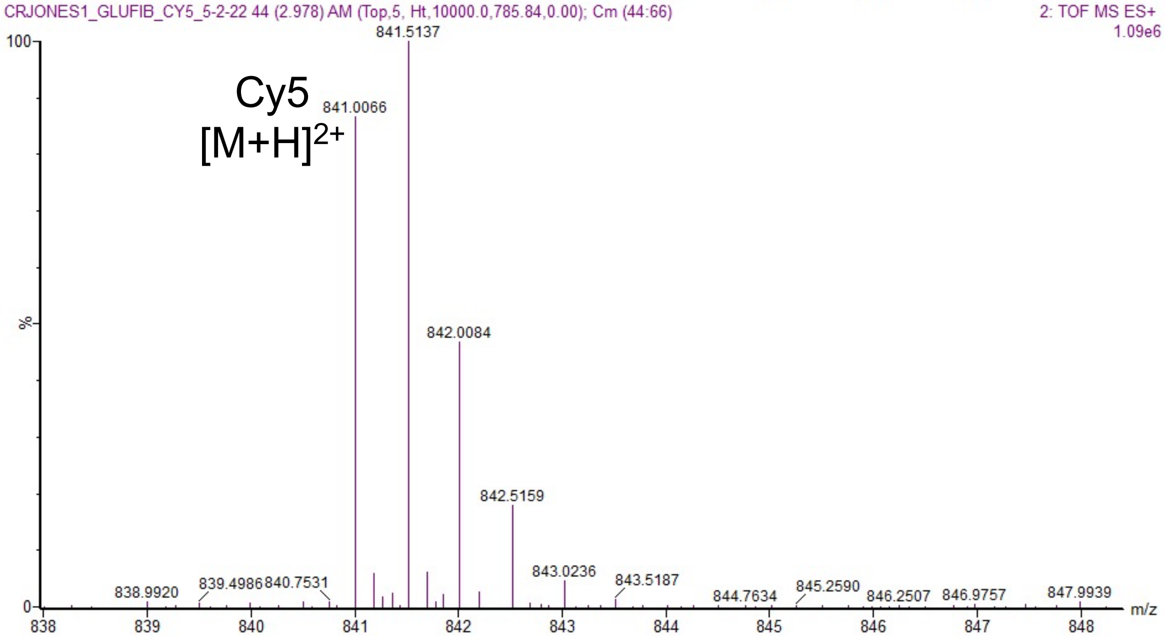

HRMS on Lys(Cy5)$_{10}$-teixobactin was performed on a Waters Synapt G2 using [Glu1]-Fibrinopeptide B as the calibrant.

Lys$_9$,Arg$_{10}$-teixobactin

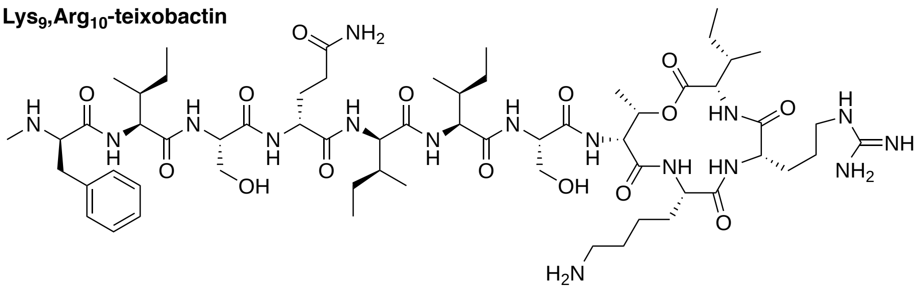

Chemical Formula: $C_{61}H_{104}N_{16}O_{15}$
Exact Mass: 1300.79

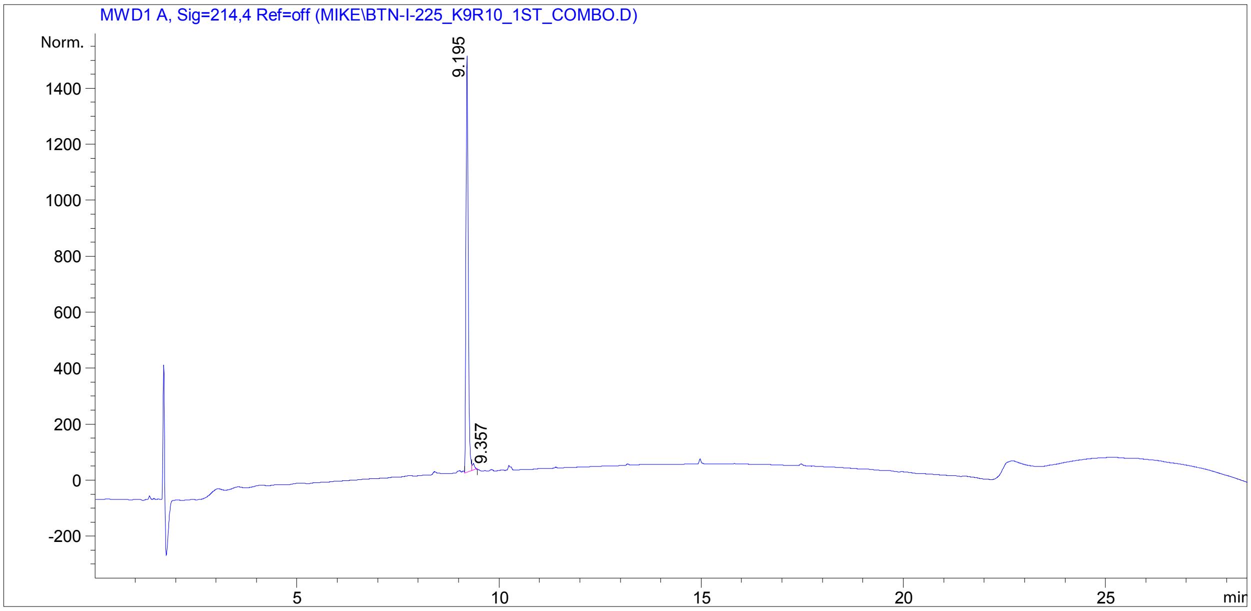


```
Peak RetTime Type  Width     Area      Height     Area
  #   [min]        [min]   [mAU*s]     [mAU]        %
----|-------|----|-------|----------|----------|--------|
   1   9.195 MF    0.0632 5099.28418 1345.13147 98.5080
   2   9.357 FM    0.0634   77.23338   20.30481  1.4920
```

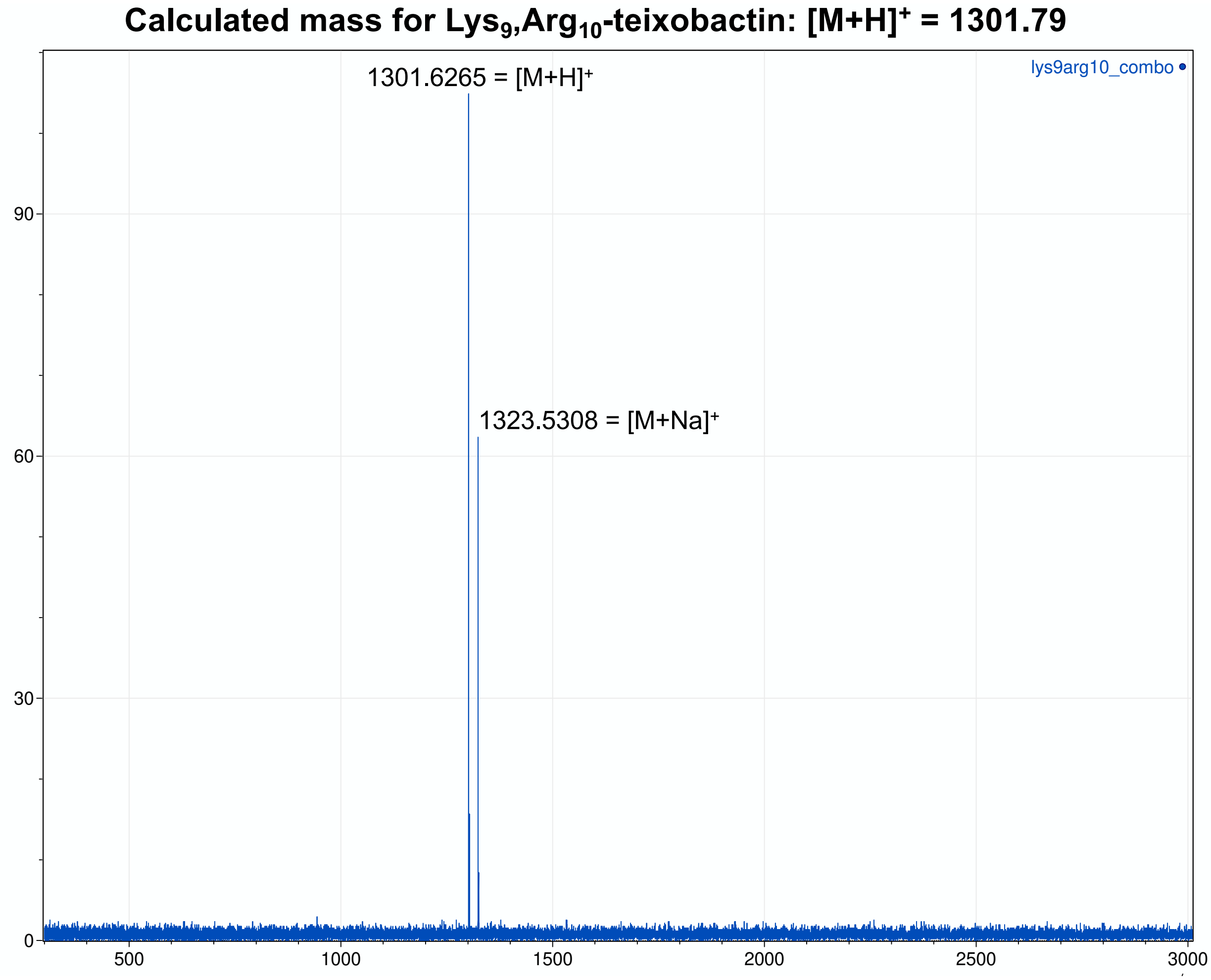
Calculated mass for Lys9,Arg10-teixobactin: [M+H]+ = 1301.79
1301.6265 = [M+H]+
1323.5308 = [M+Na]+
lys9arg10_combo
90
60
30
0
500
1000
1500
2000
2500
3000

Calculated mass for Lys_9,Arg_{10}-teixobactin: $[M+H]^+$ = 1301.79

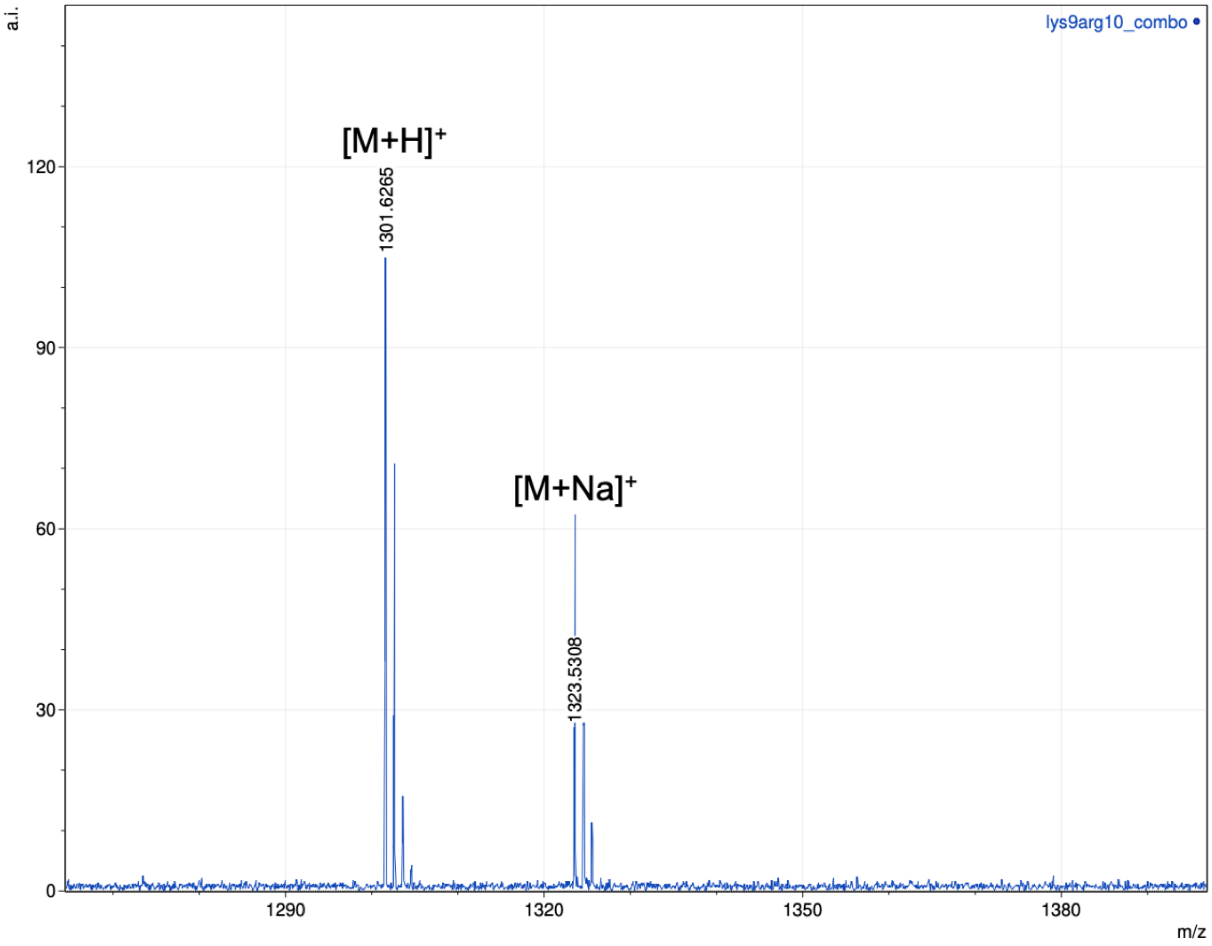

Lys(BDY FL)$_9$,Arg$_{10}$-teixobactin

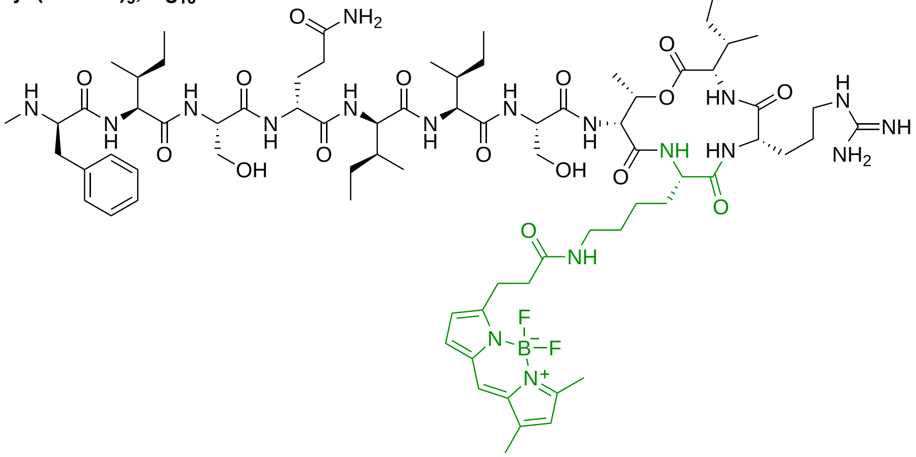

Chemical Formula: $C_{75}H_{117}BF_2N_{18}O_{16}$
Exact Mass: 1574.90

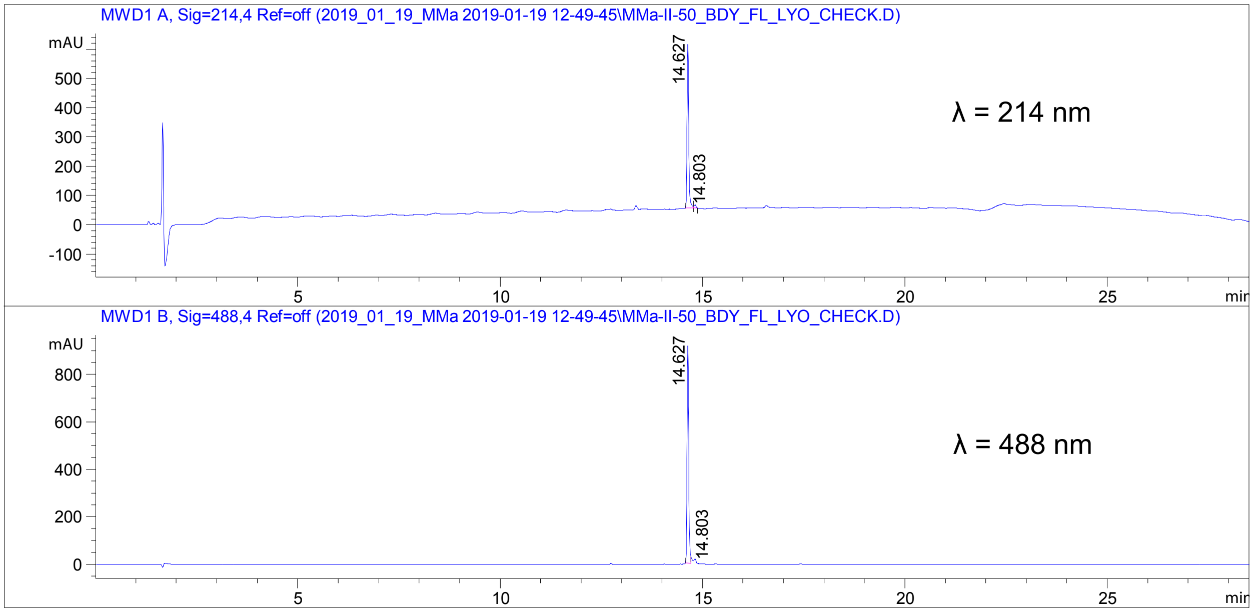

Peak #	RetTime [min]	Type	Width [min]	Area [mAU*s]	Height [mAU]	Area %
1	14.627	MF	0.0504	1718.19421	568.64935	97.9572
2	14.803	FM	0.0538	35.83104	11.09284	2.0428

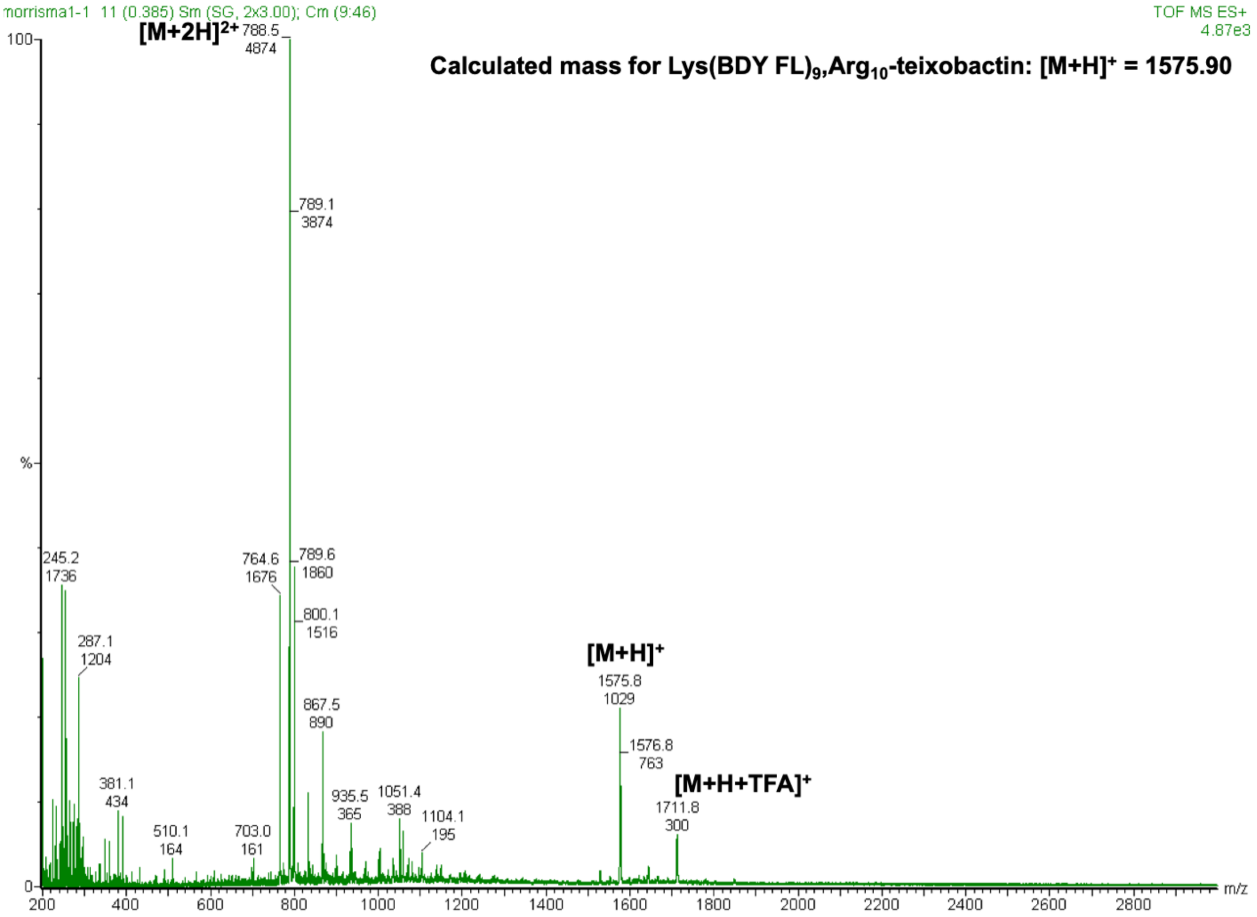
morrisma1-1 11 (0.385) Sm (SG, 2x3.00); Cm (9:46)
TOF MS ES+
4.87e3
Calculated mass for Lys(BDY FL)9,Arg10-teixobactin: [M+H]+ = 1575.90
[M+2H]2+
788.5
4874
789.1
3874
245.2
1736
764.6
1676
789.6
1860
800.1
1516
287.1
1204
867.5
890
[M+H]+
1575.8
1029
1576.8
763
[M+H+TFA]+
1711.8
300
381.1
434
935.5
365
1051.4
388
1104.1
195
510.1
164
703.0
161
100
%
0
m/z

Lys(Cy3)$_9$,Arg$_{10}$-teixobactin

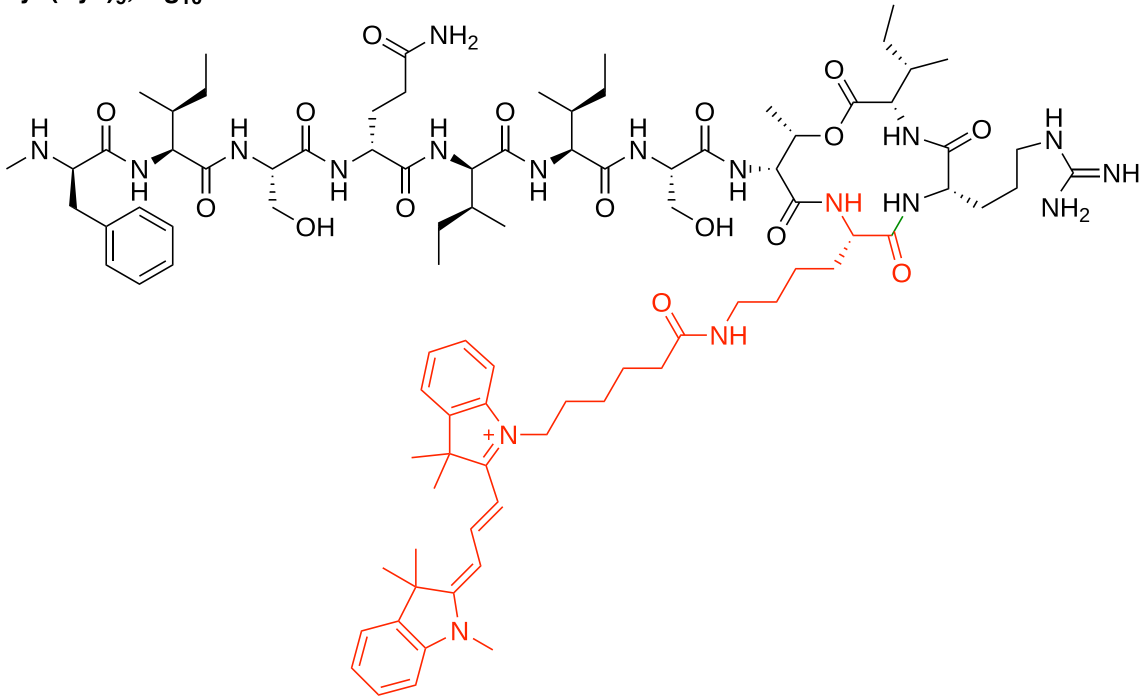

Chemical Formula: $C_{91}H_{139}N_{18}O_{16}^{+}$
Exact Mass: 1740.06

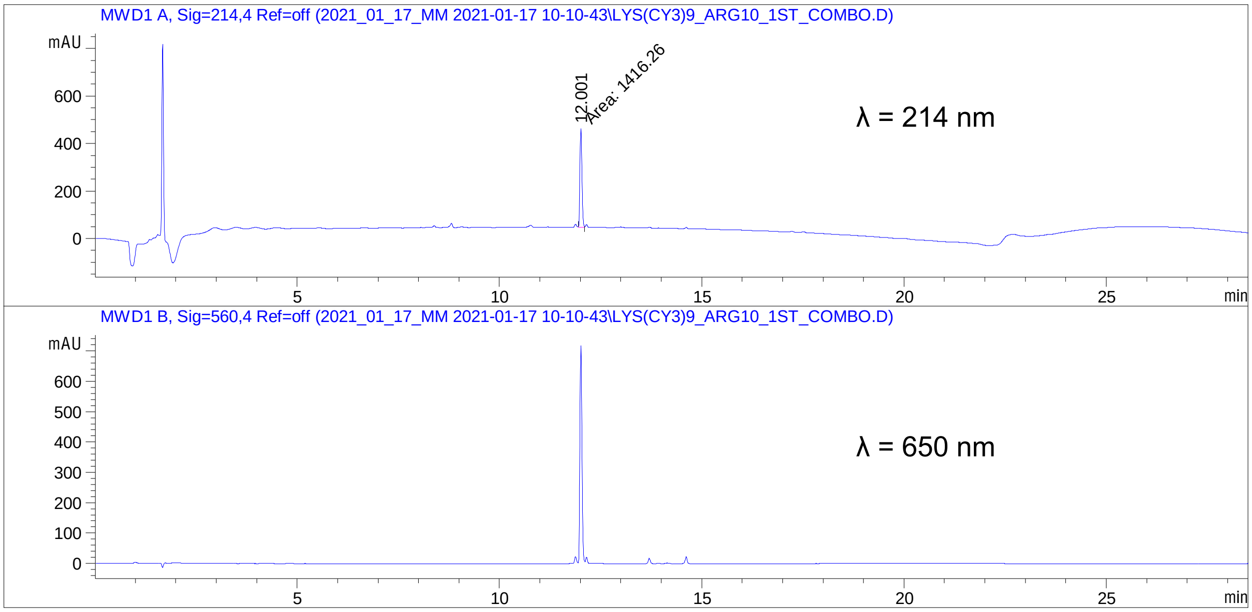

Peak #	RetTime [min]	Type	Width [min]	Area [mAU*s]	Height [mAU]	Area %
1	12.001	MM	0.0563	1416.26440	419.17258	100.0000

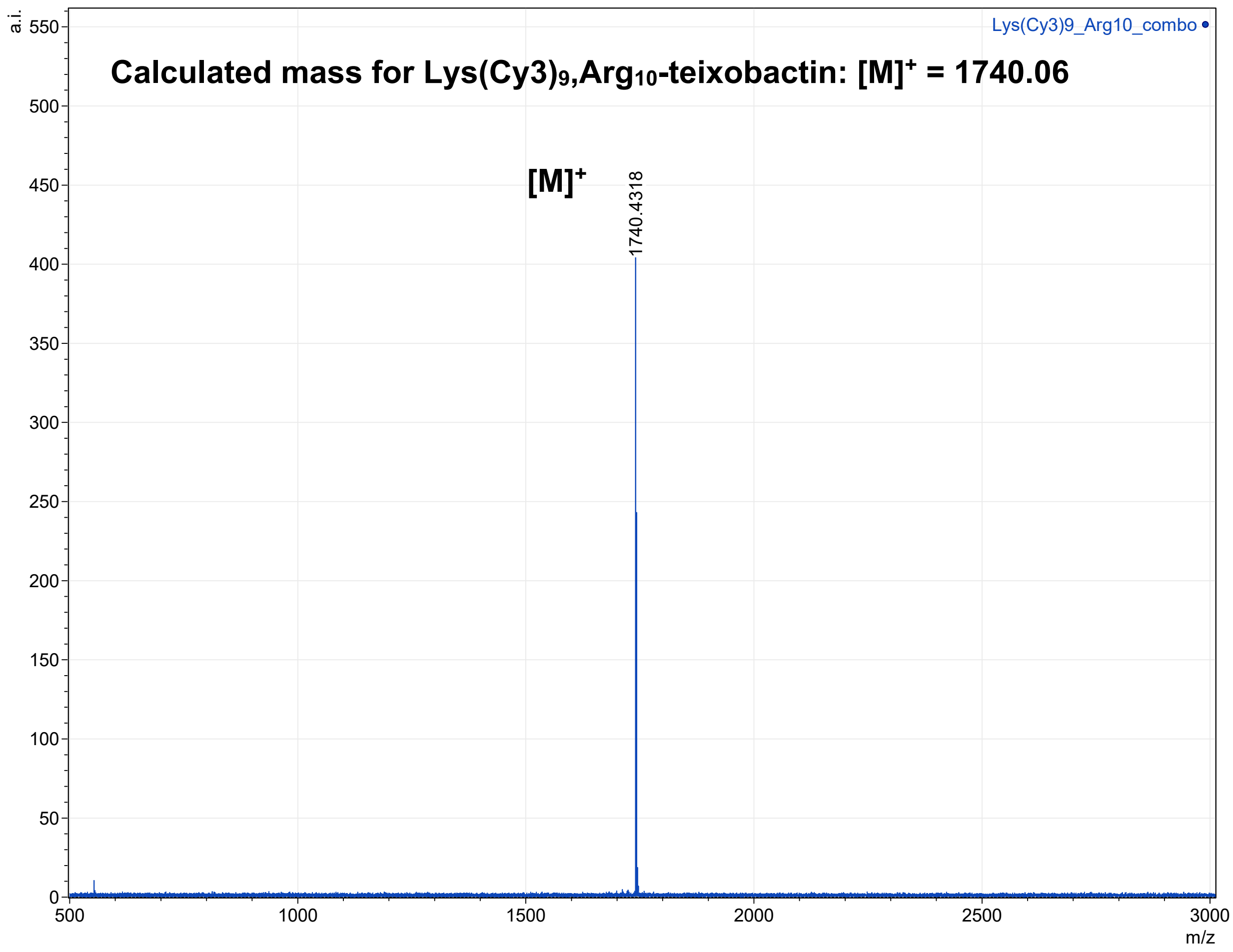
Calculated mass for Lys(Cy3)9,Arg10-teixobactin: [M]+ = 1740.06
Lys(Cy3)9_Arg10_combo
[M]+
1740.4318
a.i.
m/z
0
50
100
150
200
250
300
350
400
450
500
550
500
1000
1500
2000
2500
3000

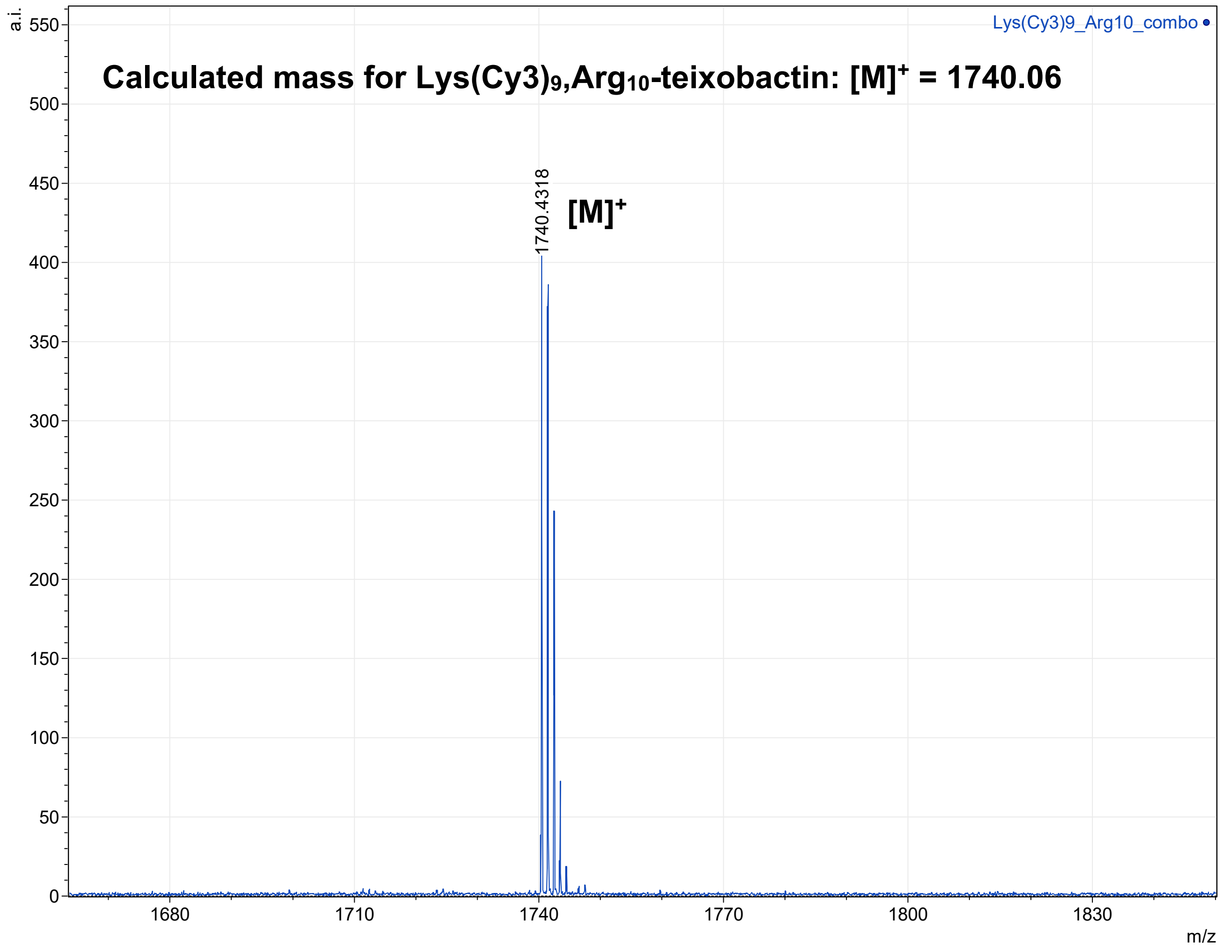

Lys(Cy3)9_Arg10_combo
Calculated mass for Lys(Cy3)9,Arg10-teixobactin: [M]+ = 1740.06
1740.4318
[M]+
a.i.
550
500
450
400
350
300
250
200
150
100
50
0
1680
1710
1740
1770
1800
1830
m/z

Lys(Cy5)$_9$,Arg$_{10}$-teixobactin

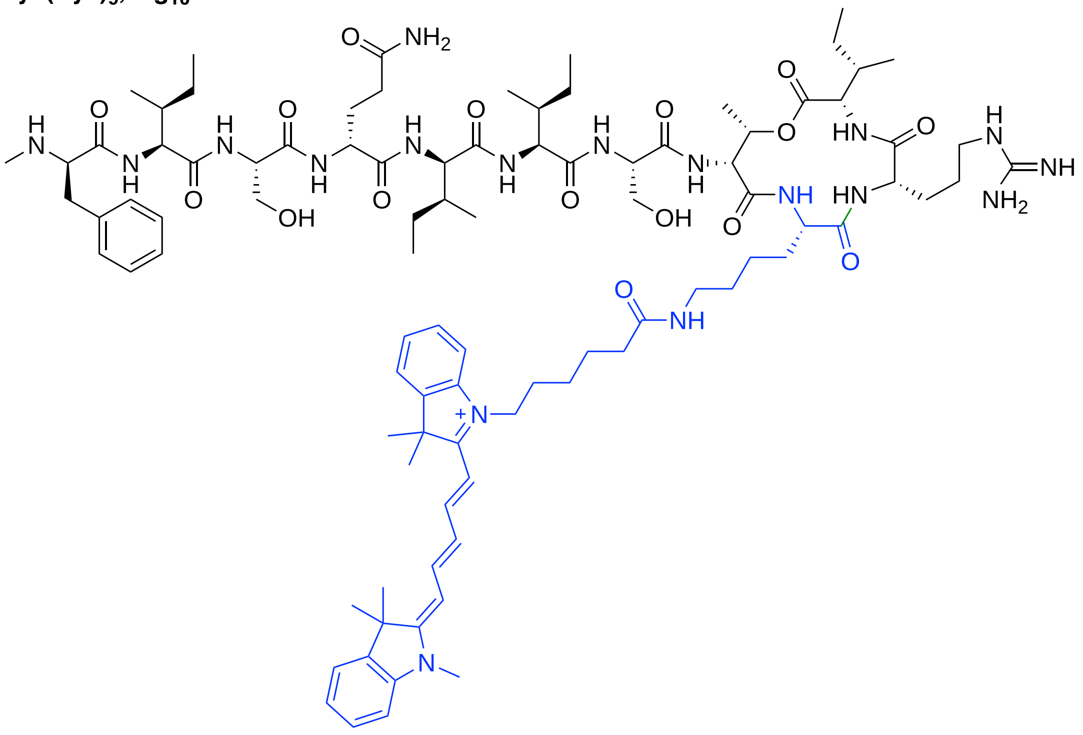

Chemical Formula: $C_{93}H_{141}N_{18}O_{16}^+$
Exact Mass: 1766.08

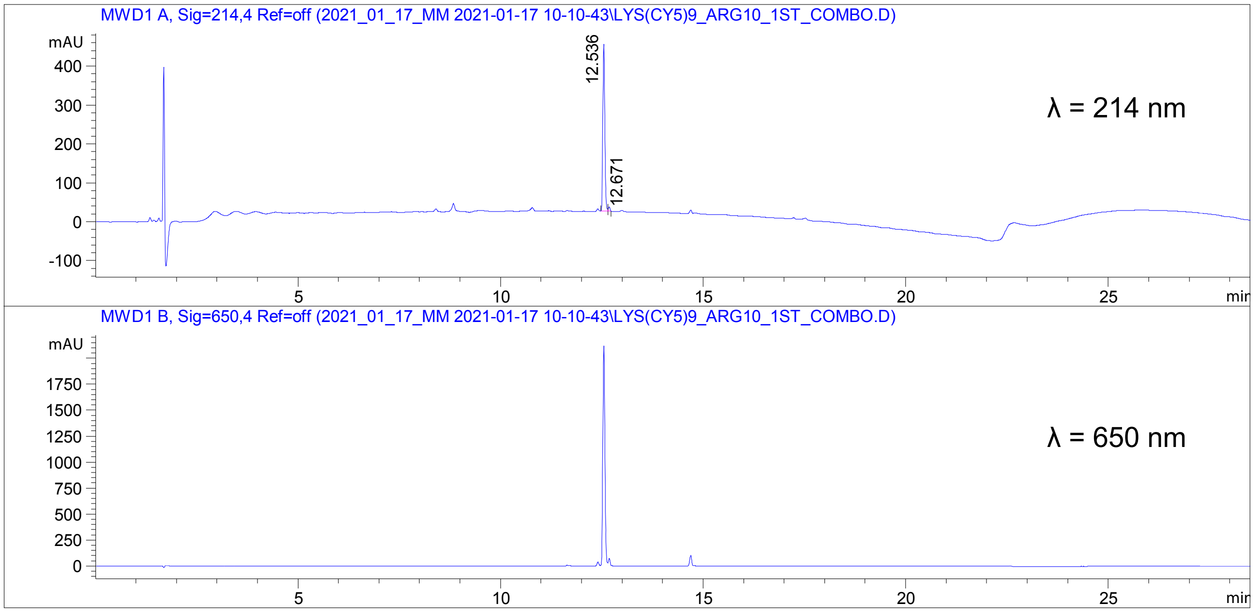

Peak #	RetTime [min]	Type	Width [min]	Area [mAU*s]	Height [mAU]	Area %
1	12.536	MF	0.0567	1469.40051	431.83197	97.8554
2	12.671	FM	0.0480	32.20267	11.17171	2.1446

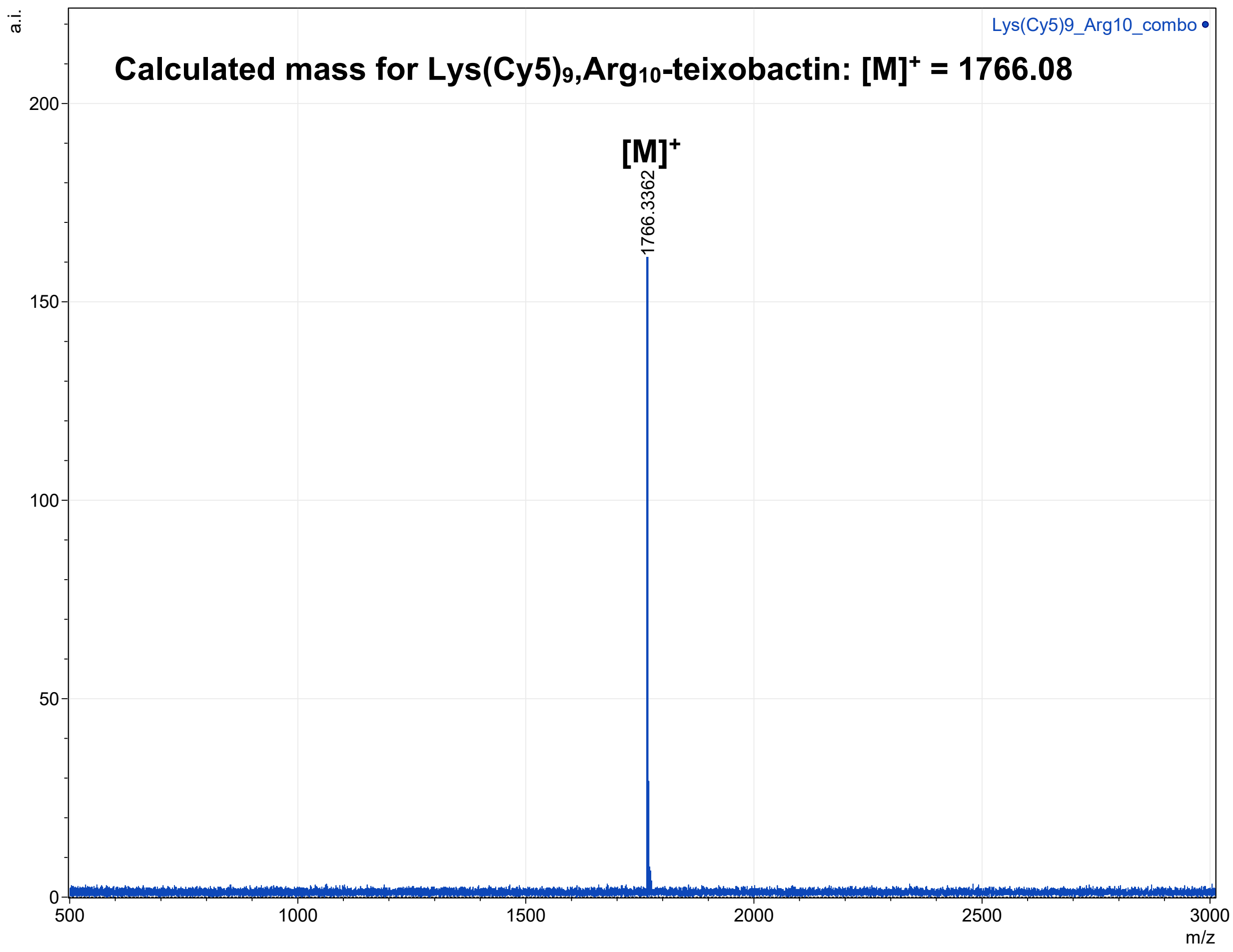
Lys(Cy5)9_Arg10_combo
Calculated mass for Lys(Cy5)9,Arg10-teixobactin: [M]+ = 1766.08
[M]+
1766.3362
a.i.
200
150
100
50
0
500
1000
1500
2000
2500
3000
m/z

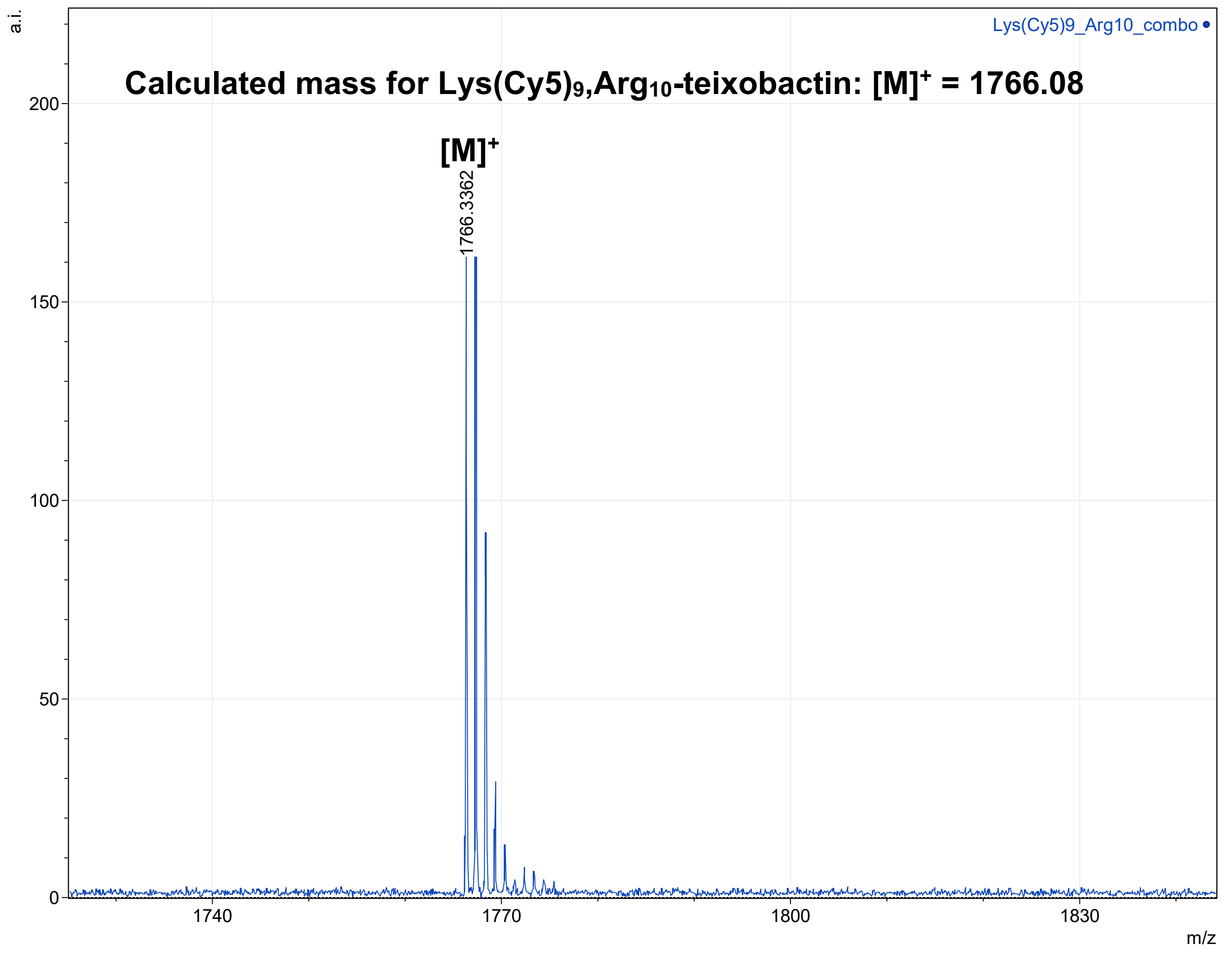

a.i.
Lys(Cy5)9_Arg10_combo
Calculated mass for Lys(Cy5)9,Arg10-teixobactin: [M]+ = 1766.08
[M]+
1766.3362
200
150
100
50
0
1740
1770
1800
1830
m/z
